# Roles of non-specific lipid transfer proteins in plant defense: structural and functional perspectives

**DOI:** 10.3389/ffunb.2025.1640465

**Published:** 2025-09-16

**Authors:** John E. McLaughlin, Nilgun E. Tumer

**Affiliations:** Department of Plant Biology, Rutgers University, New Brunswick, NJ, United States

**Keywords:** non-specific lipid transfer proteins (nsLTPs), lipid biology, plant disease resistance, plant immunity, antimicrobial peptides (AMPs), genetic engineering, lipid signaling, lipidomics

## Abstract

Non-specific lipid transfer proteins (nsLTPs) are vital and versatile components of plant cellular systems. They are characterized by a conserved eight-cysteine motif and are increasingly recognized for their dual roles in direct defense and stress modulation. nsLTPs serve critical structural and signaling functions in plant immunity. In contrast, other lipid transfer proteins, which lack the conserved cysteine motif, are primarily localized at membrane contact sites, specialized inter-organelle junctions that act as central hubs for lipid trafficking and signaling. This review explores the diverse roles of nsLTPs from structural, functional, and evolutionary perspectives, and examines current classification methodologies for the plant nsLTP superfamily. Functionally, nsLTPs contribute to the formation of protective barriers by transporting cutin monomers and other lipids, while also possessing lipid-specific antimicrobial properties that disrupt pathogen membranes. They support redox balance by scavenging reactive oxygen species, thereby minimizing oxidative stress. Additionally, nsLTPs are involved in defense signaling by transporting lipid-derived molecules essential to systemic acquired resistance. Their structural adaptability enables binding to a wide range of lipid species, underpinning their involvement in cuticle integrity, immune responses, and abiotic stress tolerance. These attributes position nsLTPs as promising targets for engineering durable, broad-spectrum disease resistance in crops. However, significant knowledge gaps remain regarding their structure-function relationships, lipid transport mechanisms, and roles in defense signaling and pathogen resistance. Addressing these challenges through advanced molecular and genetic tools could unlock the potential of nsLTPs to enhance crop resilience and contribute significantly to global food security.

## 1 Introduction

Lipid-transfer proteins (LTPs) mediate non-vesicular lipid transport, ensuring the optimal distribution of various lipids essential for cellular function, metabolism, and signaling (Chiapparino et al., 2016; Lev, 2010; Reinisch and Prinz, 2021; Neuman et al., 2022). LTPs were initially identified in the 1970s as small proteins that facilitate phospholipid transfer between membranes *in vitro*. This mode of transport is crucial for the proper distribution of lipids across the various membranes within a cell and between organelles (Lahiri et al., 2015; Wong et al., 2019, 2017). Initial LTP studies aimed to elucidate how these proteins contribute to fundamental biological processes related to lipid transport and metabolism. *In vitro* studies showed that LTPs could transfer lipids between lipid vesicles (Wirtz and Zilversmit, 1968; McMurray and Dawson, 1969; Wirtz, 1974). Molecular and structural biology research in yeast and in mammalian systems has revealed details of LTP transport that helps better understand sporulation and mitochondrial stress (Gao and Yang, 2018; Neiman, 2024; Shiiba et al., 2025). Plant research has advanced by investigating how LTPs use non-vesicular transport to control lipid homeostasis and signaling (Wang et al., 2012; Hurlock et al., 2014; de Oliveira Carvalho and Gomes, 2007).

Plants utilize a complex network of defense mechanisms to combat pathogen attack (Dodds et al., 2024; Shah and Chaturvedi, 2009; Staskawicz, 2001). Central to this defense system are lipid-mediated processes, with lipids serving as structural components, signaling molecules, and precursors for defense compounds (Kuźniak and Gajewska, 2024). Plant pathogens have evolved to manipulate host plants to promote successful infection and disease development (Shah and Chaturvedi, 2009; Christensen and Kolomiets, 2011). In response, plants have co-evolved intricate defense mechanisms to counteract pathogens, highlighting the ongoing evolutionary arms race between plants and their pathogens (Jones and Dangl, 2006). The role of LTPs has evolved from being perceived merely as lipid shuttles to key players in plant immunity (Kader, 1996; Hamaï and Drin, 2024; Cavaco et al., 2021).

Non-specific lipid transfer proteins (nsLTPs) are a subset of LTPs classified as pathogenesis-related proteins (PR-14), characterized by their conserved eight-cysteine motif (8CM) (José-Estanyol et al., 2004; Van Loon et al., 2006) which is largely confined to plant proteins, as searches have not revealed the complete 8CM outside the plant kingdom (Edstam et al., 2011). nsLTP-like proteins have been identified in *Acinetobacter baumannii* and *Paenibacillus* sp. and were considered possible cases of horizontal gene transfer (HGT) (Santos-Silva et al., 2023). nsLTPs are classified as pathogenesis-related (PR) proteins due to their induced expression in response to pathogens, abiotic factors such as cold/drought/heat, and stress-inducing chemicals like H_2_O_2_ (Sels et al., 2008; Santos-Silva et al., 2023; Van Loon and Van Strien, 1999; Liu et al., 2015; Kader, 1996).

Historically, nsLTPs were linked to cuticular barrier formation, seed development, and responses to abiotic stresses such as drought and salinity due to their ability to bind diverse lipid molecules, influencing cell wall modifications and membrane stabilization (Thoma et al., 1993; Cavaco et al., 2021; Christensen and Kolomiets, 2011; de Oliveira Carvalho and Gomes, 2007). More recently, their role in plant defense has gained prominence. Multiple nsLTPs have been found to exhibit antimicrobial activity by disrupting pathogen membranes and are integral to signaling pathways that activate immune responses, including the recognition of pathogen-associated molecular patterns (PAMPs) and inducing the accumulation of defensive compounds such as phytoalexins, lignin, and callose (Gonçalves et al., 2024c; Missaoui et al., 2022; Edstam et al., 2011).

Research continues to uncover novel defense functions of nsLTPs. Recently, a direct link to effector triggered immunity (ETI) was discovered in rice. The *Xanthomonas oryzae* pv. *oryzae* (Xoo) avirulence protein TalAE73^PXO61^ was found to trigger ETI by activating the nsLTP *OsLTPL23*. Xoo is a causal agent of rice bacterial blight (BB), a major rice disease. The TalAE73^PXO61^ effector, a transcription activator-like effector (TALE), binds to an effector binding element (EBE) located in the promoter region of the *OsLTPL23* gene in the rice. *OsLTPL23* expression was linked to ROS levels, nitrate uptake, and SA homeostasis. This represents the first documented instance of a bacterial effector protein directly targeting a plant nsLTP to trigger ETI (Jia et al., 2025). Another novel function of nsLTPs was found recently when a cowpea (*Vigna unguiculata*) nsLTP (LTP1) was found to interact with the cowpea mosaic virus (CPMV)-encoded cysteine protease 24KPro, interfering with viral replication and boosting resistance to the virus (Ji et al., 2024).

nsLTPs have also been found to contribute to systemic acquired resistance (SAR) by mediating the transport of lipid-derived signaling molecules, orchestrating whole-plant defense strategies (Carella et al., 2017; Champigny et al., 2013; David et al., 2021; Lascombe et al., 2008). Advances in genomics and proteomics have uncovered extensive nsLTP family diversity, suggesting specialized functions and an evolutionary arms race with pathogens (Edstam et al., 2011; Jang et al., 2008; Amador et al., 2021). Their conserved structural features, such as disulfide bonds linking cysteine residues, afford stability or resistance to degradation/denaturation under stress, underscoring their defensive roles. nsLTPs are crucial plant defense proteins of significant interest in agricultural biotechnology (Cheng et al., 2004; Iqbal et al., 2023; Cammue et al., 1995; Gao et al., 2022; García-Olmedo et al., 1995). Understanding the importance of nsLTPs, both historically and with modern bioinformatic tools, enhances our knowledge of plant immunity and facilitates engineering disease-resistant crops by manipulating nsLTP expression.

## 2 Structure of the review

This review examines the current understanding of nsLTPs in plant disease resistance, exploring their structural features, mechanisms of action, and involvement in defense pathways and stress homeostasis. The structure and classification of nsLTPs are presented, with an emphasis on the highly conserved eight-cysteine motif (8CM) that is critical for lipid binding. This review distinguishes between nsLTPs and other LTPs; classical LTPs are more established in their role in lipid remodeling, while the unique characteristics and functions of nsLTPs in plant defense are still being elucidated.

We examine the multifunctional roles of nsLTPs in plant defense, including antimicrobial activity, direct and indirect involvement in reactive oxygen species (ROS) scavenging, the ability to bind to fungal chitin, and participation in SAR. In this context, it’s important to highlight the emerging role of membrane contact sites (MCS) in lipid transfer. MCS are crucial for maintaining cellular homeostasis and potentially play distinct roles in plant-pathogen interactions (Michaud and Jouhet, 2019; Paul and Tiwari, 2023; Yuen et al., 2021; Lahiri et al., 2015). While LTPs, distinct from nsLTPs, are known to be associated with MCS and function in lipid exchange between organelles, the precise role of LTPs at MCS in response to biotic stress requires further investigation.

Challenges and opportunities in harnessing nsLTPs for crop protection are considered, such as addressing allergenicity and the necessity for tissue-specific targeting strategies. The functional dichotomy of nsLTPs, where certain isoforms enhance susceptibility to specific fungal pathogens while others confer resistance, necessitates mechanistic investigations to elucidate the underlying molecular determinants. This contrasting behavior underscores the importance of distinguishing nsLTP function from that of other LTPs, especially in the context of lipid dynamics and localization within the cell, including at MCS.

Key knowledge gaps remain. The mechanisms by which pathogens potentially counteract plant LTPs, including whether they employ virulence effectors that target these host proteins to compromise plant immunity, are not well understood. It is also unclear why structurally similar nsLTPs exhibit disparate functions or variable allergenic potential, and the precise mechanisms determining lipid binding specificity require further clarification. A more comprehensive understanding of nsLTPs’ involvement in specific processes like ROS scavenging is needed. We also recognize the need to better understand the role of LTPs in lipid remodeling, particularly at MCS, during disease, and their integration with other defense pathways.

This review offers insights into enhancing plant immunity with nsLTPs. A central theme is the importance of understanding how specific nsLTPs uniquely contribute to plant defense, and how their mechanisms and functions diverge from the established roles of LTPs, especially those at MCS, in lipid remodeling. We explore optimal strategies for deploying nsLTPs to engineer robust and sustainable pathogen resistance in crops. Understanding the complex interplay between lipids and plant-pathogen interactions, particularly the distinct roles of LTPs and nsLTPs, presents significant opportunities for advancing agricultural biotechnology.

## 3 Non-specific nature of nsLTPs

Plant nsLTPs are termed “non-specific” because they can interact with a diverse array of lipid molecules rather than a single, specific lipid species (Kader, 1996; Fleury et al., 2019; Santos-Silva et al., 2023; Amador et al., 2021). nsLTPs bind a wide variety of lipids including phospholipids, glycolipids, and fatty acids (Li et al., 2021b; Scheurer and Schülke, 2018; McLaughlin et al., 2021). This versatility in lipid binding underlies their multifaceted roles in plant defense, from maintaining cellular membrane integrity to facilitating long-distance immune signaling (Goyal and Mattoo, 2014). This class of small proteins contribute to both biotic and abiotic stress tolerance, participating in processes like membrane stabilization, cell wall organization, cuticle synthesis, and signal transduction (de Oliveira Carvalho and Gomes, 2007; Salminen et al., 2016; Goyal and Mattoo, 2014). However, “non-specificity” does not equate to a complete lack of selectivity. While nsLTPs can bind various lipids, they exhibit preferences and varying affinities, dictated by the structural properties of both the lipid molecules and the hydrophobic binding cavity within the nsLTP (Aldakhil et al., 2023; Madni et al., 2020; Han et al., 2001; Gonçalves et al., 2024a). This broad binding capability enables nsLTPs to engage in diverse physiological processes such as cuticle formation by transporting lipid-derived monomers, membrane stabilization and organization by modulating membrane lipid composition and fluidity, signal transduction as signal transducers in plant-pathogen interactions, responses to biotic and abiotic stresses including defense against pathogens, and plant growth and development, including roles in embryogenesis, reproduction, and germination (de Oliveira Carvalho and Gomes, 2007; Fleury et al., 2019; Santos-Silva et al., 2023; Amador et al., 2021).

Lipid overlay assays, employing lipids immobilized on hydrophobic membranes, are effective in revealing the diverse lipid affinities of nsLTPs (Dowler et al., 2002). For example, lipid-protein interaction assays can identify these affinities by incubating purified nsLTPs with commercially available membrane lipid strips which are available from companies like Echelon Biosciences (Catalog P-6002) (Shirey et al., 2017). A total of fifteen lipids are present on the strip, consisting of three important phosphoinositides and twelve other biologically significant lipids, including cardiolipin, cholesterol, and sphingomyelin. A western blot-like approach is taken to visualize the protein bound to the strip. For example, AtLTP4.4 binds a number of different lipids and with different affinities, the three strongest being phosphatidic acid (PA), phosphatidylinositol-4-phosphate (Ptdlns(4)P), Phosphatidylinositol (3,4,5)-trisphosphate (PtdLns(3,4,5)P3) (McLaughlin et al., 2021). A similar study working with a wheat nsLTP, *TaMs1*, which plays a role in pollen development, used lipid strips to show that orthologous proteins from rice (OsLTPg29) and maize (ZmLTPg11) are able to bind PA and several phosphoinositides (Li et al., 2021b). The authors were able to confirm and differentiate the roles of the orthologous rice and maize nsLTPs in pollen development in *Poaceae* using complementation of the male sterility phenotype of the wheat *tams1* mutant with the wildtype (*OsLTPg29* or *ZmLTPg11*) genes.

## 4 Structure and classification of nsLTPs

### 4.1 Type I vs Type II cavity structures

Initial classifications of nsLTPs were based on molecular mass and are categorized into two main types: Type I nsLTPs, which are roughly 9 kDa, and Type II nsLTPs, at around 7 kDa (Kader, 1996). nsLTPs are defined by a conserved eight-cysteine motif (8CM), crucial for their lipid-binding function (Madni et al., 2020; Wang et al., 2012; José-Estanyol et al., 2004). This 8CM, typically arranged as C-Xn-C-XnC-C-Xn-C-Xn-C-Xn-C-C (where ‘C’ is cysteine and ‘Xn’ is a variable number of amino acids), forms a hydrophobic cavity lined by hydrophobic amino acid side chains.

Classifications were later expanded upon by several bioinformatics labs (Boutrot et al., 2008; Santos-Silva et al., 2023; Wang et al., 2012; Amador et al., 2021; Fleury et al., 2019). A side-by-side comparison of the key criteria used in these evolving classification systems, from Kader (1996) to Huang et al. (2023), is provided in **Supplementary Table S1**. Boutrot et al. (2008) proposed nine types (I-IX) based on sequence similarity and cysteine spacing, with subsequent additions including groups X and XI (Liu et al., 2010). The major classification differences between Type I and Type II nsLTPs are shown in **Table 1**.

**Table 1:** Comparative structural and functional characteristics of plant nsLTP subtypes (Type I vs. Type II).

Feature	Type I nsLTP	Type II nsLTP	Key References
Molecular Weight (kDa)	˜9-10	˜7	(Kader, 1996)
Typical No. of Amino Acids	˜90-95	˜70	(Kader, 1996)
Predominant Helical Structure	4 *α*-helices (+ 3_10_-helix segment)	3 *α*-helices	(Maximiano and Franco, 2021)
Hydrophobic Cavity	Tunnel-like, larger volume	Triangular/V-shaped, smaller volume	(Liu et al., 2015)
Residue between Cys5-Cys6	Hydrophilic	Apolar (Hydrophobic)	(Fleury et al., 2019)
Example Ligand Preferences	Linear lipids (e.g., fatty acids)	Able to accommodate bulkier lipids (e.g., sterols)	(Fleury et al., 2019; Salminen et al., 2016)
Disulfide Bridge Pattern	C1-C6, C2-C3, C4-C7, C5-C8	C1-C5, C2-C3, C4-C7, C6-C8	(Wang et al., 2012, Liu et al., 2015)
Broad functional classification	Defense, Promotion of rhizobial interactions, Cuticular wax production and deposition	Seed development, Abiotic stress, Cell wall loosening and extension, and Signaling	(Fleury et al., 2019)

The main structural, biochemical, and functional differences between Type I and Type II nsLTPs are summarized. These two categories represent the foundational classification of the nsLTP superfamily, initially distinguished by their differing molecular masses of approximately 9 kDa and 7 kDa, respectively. The comparison highlights key distinguishing features, including the number of amino acids, the architecture of the internal hydrophobic cavity, variations in disulfide bridge patterns, and their broadly assigned functional roles in plant defense and development.

nsLTPs have been found to accommodate a diverse group of lipids such as fatty acids, phospholipids, and sterols (Salminen et al., 2016; Fleury et al., 2019). nsLTPs have also been found to bind to other hydrophobic molecules. This ability to bind a variety of lipids in addition to other diverse ligands further emphasizes the versatility of nsLTPs and their involvement in a wide range of cellular processes as highlighted in **Table 2**.

**Table 2:** Catalog of molecules associated with nsLTPs.

Category	Molecules
Lipids and Lipid-Like Molecules	Phosphatidylcholine (PC), Phosphatidylglycerol (PG), Phosphatidylinositol (PI), Phosphatidylethanolamine (PE), Palmitic acid (C16:0), Stearic acid (C18:0), Oleic acid (C18:1), Linoleic acid (C18:2), Linolenic acid (C18:3), Myristic acid (C14:0) *ω*-Hydroxy fatty acids, Cutin monomers, Very long-chain fatty acids (VLCFAs), 12-Oxo-phytodienoic acid (12-OPDA), myristic acid (C14:0)
Sterols and Related Compounds	Sitosterol, Campesterol, Stigmasterol, Brassicasterol, Brassinosteroids (e.g., castasterone, brassinolide)
Secondary Metabolites	Quercetin, Kaempferol, Apigenin, Abscisic acid (ABA), Carotenoid-derived molecules
Volatiles	Petunia VOCs Benzaldehyde, Benzyl alcohol, 2-phenylethanol methylbenzoate, benzylbenzoate, Vanillin
Pathogen-Associated and Defense Molecules	Chitin fragments, Lipopolysaccharides (LPS)
Allergenic Ligands	Peach nsLTP (Pru p 3) binds to oleic acid (OLE) and phytosphingosine; wheat nsLTP (Tri a 14) binds to linoleic acid and phospholipids such as PC

A catalog of the diverse array of ligands that have been experimentally shown to associate with nsLTPs. The list highlights the “non-specific” yet versatile binding capability of nsLTPs, which extends beyond canonical lipids (like fatty acids and phospholipids) to include other hydrophobic molecules such as sterols, secondary metabolites, and even floral volatiles. This broad ligand affinity underscores the multifaceted involvement of nsLTPs in a wide range of cellular processes, from cuticle formation and defense to signaling and development.

### 4.2 Disulfide bridge patterns

Type I nsLTPs are characterized by a conserved disulfide bond pattern, typically C1-C6, C2-C3, C4-C7, C5-C8 (Wang et al., 2012; Liu et al., 2015). This arrangement underpins a single, elongated, tunnel-like hydrophobic cavity that is highly conducive to binding and transporting a single lipid molecule. Proteindocking simulations indicate that ligands often lack a preferred orientation within these cavities, with hydrophobic interactions strongly dominating the protein-ligand interface (Fleury et al., 2019).

In contrast, Type II nsLTPs exhibit a different set of disulfide linkages, commonly C1-C5, C2-C3, C4-C7, C6-C8 (Liu et al., 2015). This altered bonding leads to a markedly different internal architecture, often featuring two adjacent hydrophobic cavities or a more triangular conformation (Liu et al., 2015). These variations suggest that Type II nsLTPs may accommodate different types or multiple ligands, or bind through distinct mechanisms. Molecular dynamics (MD) simulations have revealed the importance of specific amino acid residues for binding particular fatty acids, such as myristic acid and oleic acid in Ajwain [*(Trachyspermum ammi)*] nsLTP1 (Nazeer et al., 2019). Furthermore, subtle sequence differences, particularly in loop regions like H1–H2 and H1, influence ligand binding modes, as observed between barley HvLTP1.1 and maize ZmLTP1.6 (Salminen et al., 2016). The orientation of the ligand cavity entrance differs between the two folds. In Type I fold, it’s along an axis perpendicular to the C-terminal loop, while in Type II fold, it’s approximately parallel to it (Fleury et al., 2019).

The eight cysteines form four disulfide bridges, stabilizing the compact, *α*-helical rich fold and maintaining cavity integrity, facilitating lipid encapsulation and transfer. The N-terminal signal peptides often direct nsLTPs to the extracellular apoplast via the secretory pathway, or other compartments such as the endoplasmic reticulum, mitochondria, chloroplast, or vacuoles, depending on the type of nsLTP and associated specific signal peptide (Levesque-Tremblay et al., 2009; Nishimura et al., 2008; Li et al., 2024; Santos-Silva et al., 2023).

### 4.3 Expanded classification systems for plant nsLTPs

The nsLTP superfamily has been organized into at least eleven types based on a combination of features, including molecular mass, sequence homology, the spacing of cysteines within the 8CM domains, posttranslational modifications, and subcellular localization (Edstam et al., 2011; Boutrot et al., 2008; Santos-Silva et al., 2023) as shown in **Supplementary Table S2**. These classifications align with clear structural distinctions described above. While all nsLTPs share a conserved right-handed superhelix fold, a fundamental structural dichotomy exists between the “Type I fold” and the “Type II fold.” This divergence is driven by differences in amino acid sequence and disulfide bond patterns, which result in significant variations in their 3D architecture, internal cavities, and ligand-binding characteristics (Fleury et al., 2019).

### 4.4 Bioinformatic and structural tools to study nsLTPs

The classification and understanding of nsLTPs are being revolutionized by advanced computational tools. The European Bioinformatics Institute’s InterPro database (Blum et al., 2024) is an invaluable bioinformatics resource, unifying protein classification by integrating predictive signatures from various member databases like Pfam, SMART, PROSITE, CATH-Gene3D, and SUPERFAMILY. This approach mitigates redundancy and provides crucial insights into sequence conservation and predicted features. For instance, InterPro’s largest nsLTP family, “Bifunctional inhibitor/plant lipid transfer protein/seed storage helical domain” (IPR016140), comprises over 43,000 proteins, 362 domain architectures, and over 2,000 taxons. It also integrates data from 75 PDB solved structures and over 27,000 AlphaFold 3 predicted structures, partly based on PROSITE DOC (PDOC00516) and the PLANT LTP signature (PS00597): [LIVM]-[PA]-x(2)-C-x(1,2)-[LIVM]-x(1,2)-[LIVMST]-x-[LIVMFY]x(1,2)-[LIVMF]-[STRD]-x(3)-[DN]-C-x(2)-[LIVM].

Beyond broad databases, custom scripting, particularly in Python within the Anaconda environment (Anaconda, 2016), offers unparalleled flexibility for detailed sequence analysis. This enables researchers to precisely identify and categorize disulfide bond linkages (e.g., C1-C6, C2-C3, C4-C7, C5-C8 for Type I versus C1-C5, C2-C3, C4-C7, C6-C8 for Type II nsLTPs) in a flexible and scalable manner in addition to looking for novel patterns. For instance, by extending the 8CM spacing from n = 8–30 to n = 8–50, a new class of nsLTPs was identified in algae (Huang et al., 2023). Algal nsLTPs have divergent 8CM spacing and binding pocket residue property differences compared to land plants.

The use of the RapGreen tool developed by the de Lamotte research group was used to organize the nsLTPs superfamily into an interactive phylogenetic tree which and included phylogenetic, structural, and both Plant Ontology (PO) and Gene Ontology (GO) information (Fleury et al., 2019; Dufayard et al., 2021). The RapGreen phylogenetic analysis of 797 nsLTPs can be explored using the following website https:// phylogeny.southgreen.fr/treedisplay/index.php?data=msdmind . The seven broad nsLTP classes in this phylogenetic tree range include Type I (409 nsLTPs) and Type II (118 nsLTPs) and range to Type VIII nsLTP. This type of analysis can be applied to new nsLTPs which are identified in protein databases and with advanced deep and machine learning tools (e.g., UniProt, RCSB Protein Data Bank (RCSB PDB), and AlphaFold). Frequently Aligned Symbol Tree (FAST) and Structural Trace Analysis (STD), revealed additional information about amino acid residues that might confer functional specificity in defense but require additional study to connect with protein function (Fleury et al., 2019).

The advent of AlphaFold for highly accurate 3D protein structure prediction (Jumper et al., 2021) has provided an unparalleled dataset of predicted structures, complementing laborious experimental methods. Tools like the RCSB PDB’s Pairwise Structure Alignment tool (Bittrich et al., 2024), utilizing algorithms such as TM-align (Zhang and Skolnick, 2005), efficiently compare predicted and experimentally determined structures. This allows for robust identification of structural homologs, revealing subtle variations in folds, loop regions, and cavity architectures that refine existing classifications and can identify novel structural subgroups (Gonçalves et al., 2024a; Morales-Quintana et al., 2024). As an example of this, we downloaded the 12,880 proteins (in FASTA format) classified in the Plant non-specific lipid-transfer protein (IPR000528) group from InterPro and removed the 981 redundant sequences using FASTA file manipulation tool seqkit2 (Shen et al., 2024). A python script (McLaughlin, 2025) was used to identify 8CM motifs revealing 11,861 individual proteins with one 8CM domain, 35 proteins with two 8CM domains (UniProt format: A0A067JWZ8, A0A0D3HHF7, A0A251VQ94, A0A498ILS8, A0A498J7V5, A0A4D6NFG6, A0A4S8IBN1, A0A4Y1RRI6, A0A5B6W1L1, A0A6P5FSB9, A0A7J6HF70, A0A7J6HFY0, A0A7J7KVB4, A0A7J7MH43, A0A803MFK2, A0A834TCD7, A0A835KDA7, A0A835KME6, A0A8J5YXI7, A0A8J5ZEW1, A0A9D3VEA1, A0A9D5CJ42, A0A9E7F1U6, A0A9E7F2M4, A0A9J5YS56, A0A9Q0F1H8, A0AA38W3R9, A0AA88R1U2, A0AAD5D2I7, A0AAD6QXP8, A0AAD6WBZ6, A0AAP0LW68, A0AAQ3STU6, A0AAV7H7B0, and A0ABD3GH35), two proteins with three 8CM domains (A0AAD8S0M5, A0A6N2MXE4), and one protein with four 8CM domains (A0A0D3HQM8). Multi 8CM domain proteins have been detected before within the nsLTP family (Edstam et al., 2011). In that work, four proteins (MpLTPg2 from *Marchantia polymorpha* and PpLTPg1, PpLTPg5, PpLTPj5 from *Physcomitrella patens*) were found to have two 8CMs, while one protein (PpLTPg7 from *P. patens*) was detected with three 8CMs.

**Figure 1:**
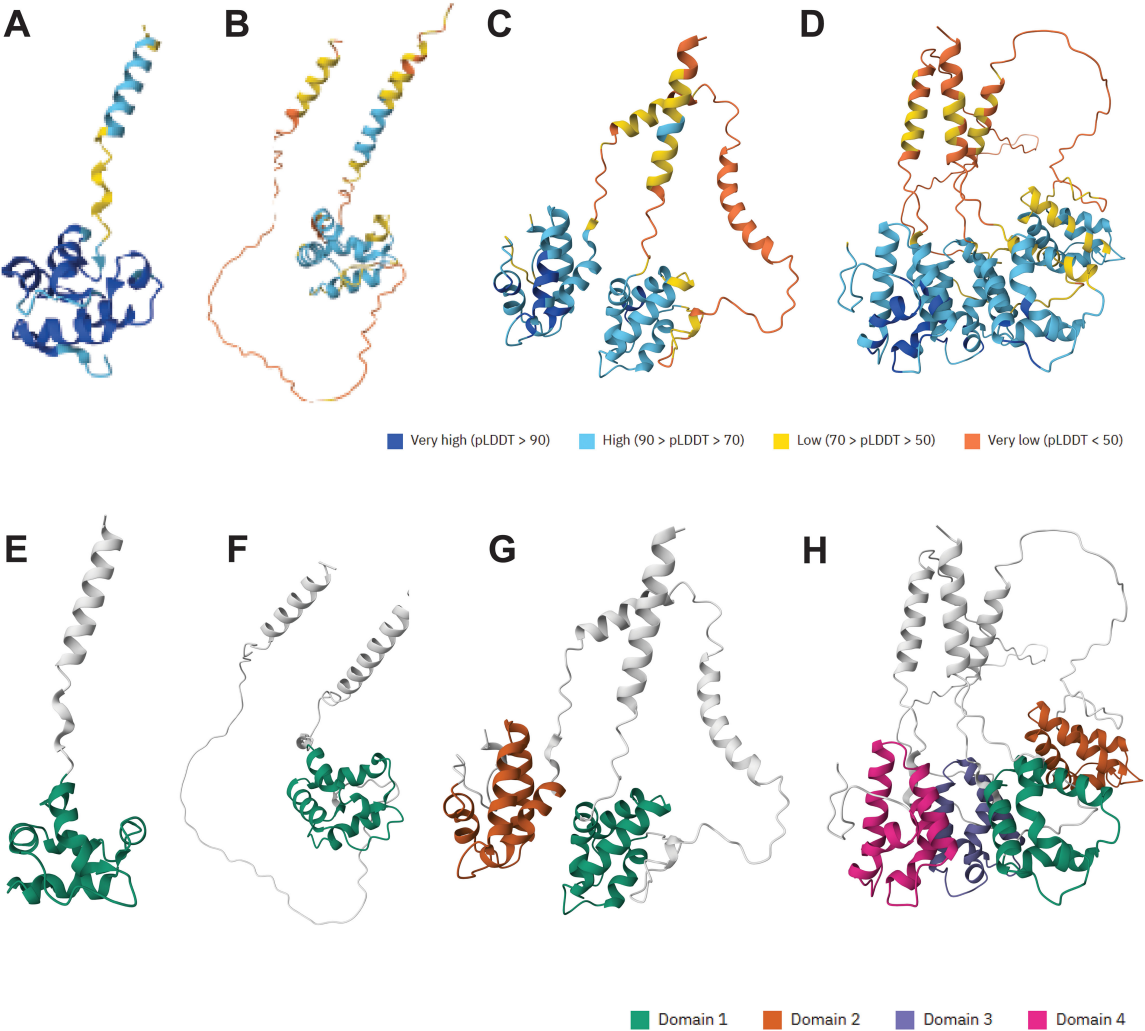
The structural diversity and novel multi-domain architecture of nsLTPs from African wild rice (*Oryza barthii*), is illustrated based on AlphaFold3 predictions. The top row **(A-D)** shows the 3D structures colored by their predicted Local Distance Difference Test (pLDDT) confidence scores, while the bottom row **(E-H)** highlights the functional eight-cysteine motif (8CM) domains as classified by The Encyclopedia of Domains (TED). The panels display a progression from a typical single 8CM domain nsLTP [**(A, E)**; A0A0D3GPP4] and a GPI-anchored variant [**(B, F)**; A0A0D3FEW3], to the novel discovery of an nsLTP with two 8CM domains [**(C, G)**; A0A0D3HHF7] and a particularly novel protein possessing four 8CM domains [**(D, H)**; A0A0D3HQM8]. The identification of these multi-domain proteins significantly expands the known structural variety within the nsLTP superfamily.

A bioinformatic analysis of the UniProt database using a custom Python script identified two novel multidomain nsLTPs in African wild rice (*Oryza barthii*). These proteins, A0A0D3HHF7 and A0A0D3HQM8, were found to possess two and four eight-cysteine motif (8CM) domains, respectively. AlphaFold3 structures are available for these novel proteins. For comparison, the AlphaFold3 structures for a single 8CM domain (A0A0D3GPP4) (A,E) and a GPI-anchored, single 8CM domain (A0A0D3FEW3) (B,F), a two 8CM domain (A0A0D3HHF7) (C,G), and a four 8CM domain protein (A0A0D3HQM8) (D,H), all from *Oryza barthii*, are shown in **Figures 1A–D**, which represent AlphaFold3 predicted structures with predicted Local Distance Difference Test (pLDDT) scores. The pLDDT score is a per-residue confidence metric that indicates how closely a predicted protein structure is expected to match its experimentally determined 3D structure. **Figures 1E–H** represent the same structures but highlighting a domain defined by The Encyclopedia of Domains (TED). The TED domain represents a systematically identified and classified protein domain within the AlphaFold Protein Structure Database (Lau et al., 2024). To confirm lipid binding activity of these novel proteins, an assay using recombinant versions of these proteins challenged against PIP (phosphoinositides) strips could be used to identify potential protein-lipid interactions (Shirey et al., 2017). The nsLTP classification systems are summarized in **Table 3**.

**Table 3:** Evolution of plant nsLTP classification criteria.

Reference/Approach	No. of Types/Groups	Key Defining Criteria	Range MM (kDa)	pI Range	8CM Notes & Other Structural/Computational Aspects
Traditional Classification Systems					
Kader, 1996	2 (Type I, II)	Molecular mass	Type I (9), Type II (7); Usually 8.5–12	Not consistently summarized	C-Xn-C-Xn-CC-Xn-CXC backbone; different Cys pairing for Types I and II.
Boutrot et al., 2008	9 (I–IX)	Sequence similarity; 8 Cys intervals	8.9–12.3 (Type I), 6.9–8.1 (II), 6.7–6.8 (III), 3.9–12.2 (IV), etc.	Mostly basic (3.9–12.7)	Consensus C-Xn-C-Xn-CCXn-CXC-Xn-C-Xn-C; spacing variations per type.
Wang et al., 2012	5 (I–V) + Type X	Conserved Cys residue patterns; sequence similarity	7.1–12.1 (I), 7.8 (II), 7.8–10.7 (III), etc.	Not summarized	Prosite-style patterns; flanking residues detailed elsewhere.
Edstam et al., 2011	10 (1, 2, C–K)	Sequence similarity, GPI anchor, intron position, Cys spacing	Types range: 4.5–15.0 depending on species	Not summarized	Cys spacing and intron positions (e.g., downstream of 8th Cys).
Fleury et al., 2019	10 + 12 unclassified	Length (60–150 aa); strict 8 Cys post-signal peptide; monodomain only	Not explicitly by type	Not summarized	Strict 8CM pattern; excludes types with <8 Cys (e.g., Boutrot VII).
Huang et al., 2023	1 new algal lineage (29 genes)	8CM presence; signal peptide; phylogenetic clustering	10.4–50.3 (mostly 10–25)	Example: CrLTP1 (5.53), CrLTP2 (8.64)	Extended Cys spacing (n = 8–50); N-terminal extension before 1st Cys.
Enhanced Classification Criteria (Computational Approaches and Validation via Recombinant nsLTP Testing Assays)					
Proposed (This Review)	Dynamic; functionally guided clusters	Integration of sequence, structure, lipid-binding predictions	Variable by sub-class	Variable by sub-class	**Involve more 3D Structure analysis:** Predicted *α*-helical bundle and disulfide bonds (AlphaFold). **Perform computational lipid binding:** Affinity and cavity properties via docking. **Test recombinant nsLTP lipid binding and direct antimicrobial testing:** PIP strip western analysis and zone of inhibition assays. **Investigate two and three 8CM domain nsLTPs:** Test if these proteins have novel lipid binding and defense capacities. **Signal Peptide:** Identify novel signal peptides beyond secretion to the apoplast **Intron Position and Size:** Further expand 8CM Pattern. **Combined 1D and 3D analysis relative to other known nsLTPs:** PDB Pairwise Structure Alignment tool.

The evolution of classification criteria for the plant nsLTP superfamily. A progression is shown from early classification systems, which were based on fundamental biochemical properties like molecular mass and sequence similarity, to more complex modern frameworks. These newer systems incorporate more detailed criteria, including the precise spacing of the eight-cysteine motif (8CM), the presence of GPI anchors, and intron positions. A proposed framework from this review, advocates for a dynamic and functionally-guided classification system that integrates advanced computational and structural tools like AlphaFold, molecular docking, pairwise structure alignment, and functional analysis to better correlate nsLTP structure with its diverse biological functions. The bolded section headings indicate proposed research areas and experimental methods for future investigations of nsLTPs.

Furthermore, the integration of advanced molecular docking programs and MD simulations (Paggi et al., 2024; Neubergerová and Pleskot, 2024) allows for atomic-level modeling of nsLTP-ligand interactions. These simulations quantify interaction energies, identify critical binding residues, and explore ligand dynamics (Fleury et al., 2019; Nazeer et al., 2019; Salminen et al., 2016). This capability is invaluable for correlating structural variations with specific ligand preferences, adding a functional dimension to structural classification. In essence, the synergistic application of comprehensive bioinformatics databases (e.g., InterPro), flexible Perl/Python scripting, AlphaFold’s structural predictions, advanced structural comparison tools (e.g., TM-align), and sophisticated molecular docking/MD simulations enables a more granular and functionally relevant classification of nsLTPs. Future nsLTP classifications can take advantage of these tools and the accumulated knowledge built up from sequence and structural information that is available in UniProt and AlphaFold databases. The PDB Pairwise Structure Alignment tool, for instance, enables the simultaneous alignment of up to 10 protein 3D structures (Bittrich et al., 2024). This provides an excellent method to compare to Type I, Type II, GPI-anchored nsLTPs, and other nsLTPs in a single tool providing alignment quality structure-based scores, such as Root-Mean-Square-Deviation (RMSD, measured in Angstroms)˚ and TM-score (ranging from 0.00 to 1.00), alongside sequence-based metrics like percent identity and the number of aligned residues. This multidisciplinary computational approach will accelerate the discovery and characterization of novel subfamilies, providing a more complete picture of their diverse roles in plant physiology and defense.

### 4.5 Relationship between nsLTP structure and defense function

The most complete discussion of the relationship between nsLTP structure and plant defense is given by the de Lamotte laboratory (Fleury et al., 2019; Dufayard et al., 2021). They utilized a comprehensive approach combining phylogenetic and structural information to classify nsLTPs and investigate their involvement in defense mechanisms. To understand nsLTP function and variability across the superfamily, researchers analyzed a large dataset of 797 nsLTP protein sequences, which included both experimental 3D structures, including X-ray crystallography, NMR spectroscopy, cryo-electron microscopy data, and computer modeling. Type I nsLTPs formed a well-supported monophyletic group and predominated, making up over half of their dataset (417 out of 797 sequences). Type II nsLTPs were the second most abundant, with 126 sequences. Using the structural information available with these sequences, the study then classified the nsLTPs into two distinct structural categories: the Type I fold and the Type II fold. As expected, phylogenetic Type I nsLTPs were consistently found to have the Type I fold.

The Type I family is the predominant group for defense-related nsLTPs, encompassing 28 proteins that have been functionally classified as defense-related. In contrast, the Type II group contained only 3 classified defense-related nsLTPs, including the known protein AtDIR1 (Q8W453). This skewed distribution strongly suggests that Type I nsLTPs play a more significant role in plant defense (Fleury et al., 2019). Further experimental validation is required to refine the functional classification of Type I and Type II nsLTPs, many of which currently lack specific GO annotations.

### 4.6 Evolutionary aspects of nsLTPs

Investigation of the evolutionary history of nsLTPs can provide valuable insights into their functional diversity and role in plant adaptation to different environments and pathogens. nsLTPs are found in all land plants and recently in green algae (Edstam et al., 2011; Edqvist et al., 2018; Huang et al., 2023). The diversity of nsLTP subfamilies is more limited in non-seed plants compared to seed plants, suggesting that new nsLTPs may have evolved during land plant evolution (Edstam et al., 2011). Phylogenetic analyses indicate that nsLTPs have undergone both tandem and segmental duplications, contributing to their functional diversity (Edstam et al., 2011; Wei et al., 2025). The adoption of novel nsLTP types likely assisted plants in adjusting to the harsh new environments and disease pressures.

Differences in nsLTPs between plant species and within families have been revealed via structural analysis and bioinformatic comparisons. For example, a survey of nsLTPs in rice and *Arabidopsis thaliana* revealed 52 rice nsLTPs and 49 Arabidopsis nsLTPs (Boutrot et al., 2008). The authors employed comparative genomics, using the identified rice nsLTPs as a basis, to identify 156 putative nsLTPs in wheat. The nsLTP gene family in maize (*Zea mays*) includes 65 genes, which can be divided into six types (1, 2, C, D, G, and a unique type X), each with distinct expression patterns and functions (Fang et al., 2023). Similarly, in *Brassica rapa*, 63 nsLTP genes were identified and grouped into nine types (I, II, III, IV, V, VI, VIII, IX, and XI), with specific roles in defense, reproduction, and stress responses (Li et al., 2014). In barley, 70 nsLTPs were classified, based on phylogeny, protein characteristics and gene structures, and placed in groups 1, 2, C, D, and G (Zhang et al., 2019). This diversity highlights the extensive expansion and functional specialization of the nsLTP family across the plant kingdom. **Supplementary Table S2** provides a comprehensive overview of the number, types, and key characteristics of nsLTPs identified in representative species, from green algae and bryophytes to angiosperms like rice and *A. thaliana*. Research into barley nsLTPs has been extensive due to the connections with the brewing industry as these proteins significantly influence key beer quality attributes such as foam stability, head retention, haze formation, and flavor stability (antioxidant capacity) during storage (Cai et al., 2019; Stanislava, 2007; Wu et al., 2011; Douliez et al., 2001).

The adaptation and evolution of nsLTPs in response to different pathogens or environments are driven by selective pressures, including those from pathogens. For instance, nsLTPs involved in pathogen defense have evolved to recognize and respond to specific PAMPs, impacting the plant’s immune response. A good example of this was shown by the work of Situ et al. (2024). The oomycete *Peronophythora litchii* secretes a pectin acetylesterase (PlPAE5), which was found to destabilize the litchi (*Litchi chinensis*) lipid transport protein (LcLTP1), reducing salicylic acid (SA) production and promoting infection. This interaction indicates that pathogens target LTPs to disrupt key plant defense signaling pathways. The interaction between PlPAE5 and LcLTP1 exemplifies a counter-defense strategy, where the pathogen interferes with the plant’s defense mechanism.

Additionally, nsLTPs have adapted to various environmental stresses, such as drought and salinity, by modulating lipid composition and signaling pathways (Xiao et al., 2023; Fang et al., 2023; Santos-Silva et al., 2023; Riahi et al., 2021). With their varied structures and evolutionary adaptations, nsLTPs play critical roles in plant defense, development, and stress responses. Investigating their molecular mechanisms and evolutionary history reveal valuable insights into their functions and potential applications in agricultural biotechnology.

## 5 Lipid transfer by nsLTPs

### 5.1 Mechanism of lipid binding and transfer

As detailed in the nsLTP structure section, the highly conserved 8CM, which forms four disulfide bridges, stabilizes nsLTPs. This stabilization is crucial for maintaining the integrity of the hydrophobic cavity, a feature that allows these proteins to bind and encapsulate lipid molecules (Wang et al., 2012). The lipid-binding process begins when lipid molecules interact with the hydrophobic cavity of nsLTPs. The internal cavity, lined with hydrophobic amino acid residues, creates an environment conducive to the stable binding of lipid molecules. Key amino acid residues involved in lipid binding include hydrophobic residues such as leucine, isoleucine, valine, and phenylalanine, which interact with the lipid tails (Madni et al., 2020). Mutational and modeling approaches have been used to define key residues for both protein stability and antimicrobial properties (Fleury et al., 2019; Gonçalves et al., 2024a).

### 5.2 Influence of hydrophobic cavity structure on lipid specificity

**Figure 2:**
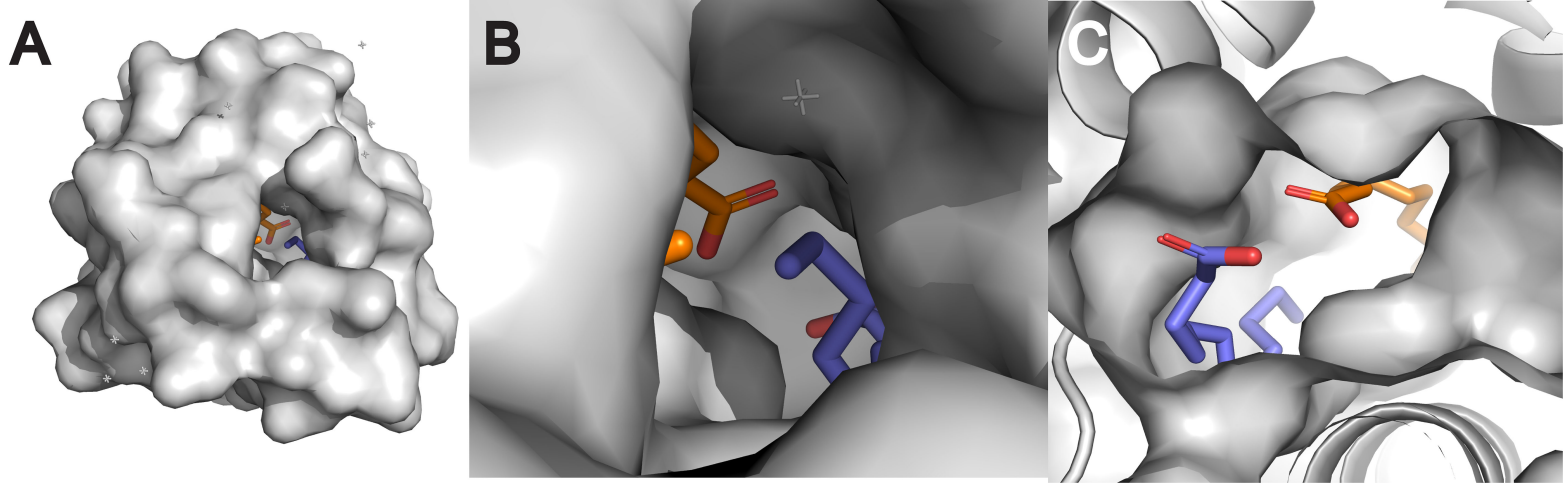
Surface structures of nsLTP from eggplant (*Solanum melongena*) (PDB ID: 7W9A) (Madni et al., 2023) visualized with PyMOL 3.1. The visualizations show the overall surface of the protein **(A)**, a magnified view into its hydrophobic cavity **(B)**, and a cross-section revealing two distinct lauric acid molecules sequestered within **(C)**. This ability to bind multiple lipids at once is a key demonstration of the structural plasticity that underlies the “non-specific” binding nature of nsLTPs.

The structure of the hydrophobic cavity significantly influences lipid specificity. The cavity’s size, shape, and flexibility determine which lipid molecules can be accommodated (Reinisch and Prinz, 2021; Missaoui et al., 2022; Sawano et al., 2008). For example, the hydrophobic cavity of nsLTPs can adjust its volume to accommodate various ligands, ranging from C10 to C18 fatty acids (Han et al., 2001; Liu et al., 2015). This structural plasticity allows nsLTPs to bind a wide array of lipid molecules, including multiple lipids simultaneously, contributing to their non-specific binding nature (Wang et al., 2012; Sodano et al., 1997; Charvolin et al., 1999; Wong et al., 2019; Douliez et al., 2001). For example, **Figure 2** presents a Pymol rendering of *Solanum melongena* nsLTP determined by X-ray crystallography which demonstrates the presence of two lauric acid molecules sequestered within the protein’s hydrophobic cavity (Madni et al., 2023).

The hydrophobic cavity’s ability to shield the hydrophobic tails of lipid molecules during transfer is essential for preventing the exposure of these tails to the aqueous cellular environment, thereby facilitating efficient lipid transport (Avula et al., 2021). The binding sites of nsLTPs exhibit such flexibility that they have even been shown to accommodate volatiles in *Petunia hybrida* (Liao et al., 2023). This research provides mechanistic insights into how hydrophobic compounds, including volatiles, cross the hydrophilic cell wall. Recent advancements have unveiled the molecular details of lipid transport by yeast LTP VPS13, providing crucial insights into its function (Adlakha et al., 2022; Melia and Reinisch, 2022; Park et al., 2016; Neiman, 2024). This work revealed that VPS13 forms a bridge containing a hydrophobic channel at membrane contact sites to facilitate bulk glycerolipid transport between organelles. This bridge-conduit model is highly relevant for plants, where proteins like ATG2 likely employ a similar mechanism to mediate the large-scale lipid flux required for processes such as autophagy-related membrane biogenesis and constitutive inter-organelle lipid homeostasis (Osawa et al., 2019; Valverde et al., 2019; McEwan and Ryan, 2022). These lessons from model organisms such as yeast provide important clues into lipid transport mechanisms of actions in plants.

### 5.3 Energetics and thermodynamics of lipid transfer

nsLTPs facilitate non-vesicular lipid transfer, which is distinct from the energy-dependent vesicular transport (Wong et al., 2017). The energetics of lipid transfer by nsLTPs involve both enthalpic and entropic contributions (Wong et al., 2019). The binding of lipid molecules to nsLTPs is primarily driven by hydrophobic interactions, which release water molecules from the hydrophobic cavity, resulting in a favorable entropic gain (Mouritsen, 2013). nsLTPs and LTPs function by reducing the energy barrier for lipids to leave bilayers, as the lipid is transferred into the hydrophobic cavity of the protein rather than into the aqueous phase (Reinisch and Prinz, 2021). The formation of disulfide bridges within the 8CM also contributes to the stability of the lipid-protein complex, providing an enthalpic contribution to the binding process (Maldonado et al., 2002). In general, metabolic energy is not required for LTPs to transfer lipids. LTPs facilitate lipid transfer by lowering the energy needed for a lipid to detach from a membrane. This occurs because the lipid enters the protein’s hydrophobic cavity rather than the less favorable aqueous environment (Reinisch and Prinz, 2021). Active lipid transport, particularly against a concentration gradient, necessitates energy. ATP-binding cassette (ABC) transporters are often involved in these processes, using ATP hydrolysis to power the movement of lipids (Christensen and Kolomiets, 2011; Roston et al., 2012; Sakuragi and Nagata, 2023; Norris et al., 2024).

## 6 nsLTPs and plant resistance

Plant resistance to pathogens relies on a multi-layered defense strategy that includes physical barriers, direct antimicrobial action, and sophisticated stress response mechanisms. nsLTPs and, more broadly, LTPs, play significant and interconnected roles in all these aspects of plant immunity (García-Olmedo et al., 1995; Gao et al., 2022; Carella et al., 2017).

The first line of defense is often the plant cuticle, a hydrophobic layer composed primarily of cutin and waxes that prevent pathogen penetration and reduce water loss (Arya et al., 2021; Zhao et al., 2020). Crucially, nsLTPs and LTPs are essential for the formation and maintenance of this barrier. They transport the precursors of cutin and waxes from their sites of synthesis within the endoplasmic reticulum (ER) to the plant surface (Jacq et al., 2017; DeBono et al., 2009; Wang et al., 2022). Specific examples include *Arabidopsis thaliana* LTPG1 and LTPG2, which transport wax precursors (DeBono et al., 2009).

Beyond their structural role in cuticle formation, nsLTPs exhibit direct antimicrobial activity against a wide range of pathogens, including fungi, bacteria, and viruses (Cammue et al., 1995; Christensen and Kolomiets, 2011; Kirubakaran et al., 2008; Avula et al., 2021). This activity stems from the inherent ability of nsLTPs to bind and transport lipids. By doing so, nsLTPs can bind to and disrupt pathogen membranes, limiting pathogen growth and spread (Diz et al., 2006; Regente et al., 2005; Chen et al., 2024). The mechanisms of antimicrobial action are diverse, including membrane permeabilization, pore formation, ion leakage, inhibition of pathogen enzymes, and disruption of crucial metabolic pathways (Melnikova et al., 2022). Notably, the antimicrobial activity of nsLTPs often shows specificity toward particular pathogens and not self, suggesting that they may recognize and target specific lipid molecules present in pathogen membranes (Terras et al., 1992; Maximiano and Franco, 2021; Cammue et al., 1995).

In addition to direct antimicrobial effects of nsLTPs, plants must balance ROS production and scavenging during pathogen attack. While ROS are produced as part of the plant’s defense response, excessive accumulation can lead to oxidative damage. nsLTPs have been found to play a role in responding to ROS stress in plants. One feature has been the discovery that nsLTPs contribute to ROS scavenging, thereby maintaining cellular redox balance under stress. This would tend to disfavor biotrophs such as the powdery mildew causing fungi (*Ascomycetes*), the *Basidiomycetes* which cause fungal rusts, and *Oomycetes* causing downy mildew diseases (Mapuranga et al., 2022) but favor resistance to fungi such as *Botrytis cinerea* and *Sclerotinia sclerotiorum* which show hemibiotrophic or necrotrophic lifestyles (Barna et al., 2012). Some nsLTPs, such as *Arabidopsis thaliana AtLTP4.4* was shown to enhance resistance to the hemibiotrophic fungal pathogen *Fusarium graminearum* (*F.g.*), possess antioxidant activity and directly scavenge ROS (McLaughlin et al., 2015, 2021). In *Brassica napus*, LTP-II was found to function as a ROS scavenger and antioxidant in guard cells, as evidenced by *ltp-II* mutant plants showing higher ROS and lower free thiols after flg22 treatment. Furthermore, treating *ltp-II* mutants with the ROS scavenger catalase restored stomatal aperture differences, confirming the role of *LTP-II* in mitigating oxidative stress (Balmant et al., 2021). nsLTPs have also been found to negatively regulate resistance by impacting the abundance of H_2_O_2_ and reducing the hypersensitive response (HR) to fungal infection. Virus-induced gene silencing (VIGS) of wheat ortholog of the *Arabidopsis DEFECTIVE IN INDUCED RESISTANCE 1* (*DIR1*) gene, *TaDIR1–2* significantly increased H_2_O_2_, SA, and resistance to the biotrophic stripe rust pathogen, *Puccinia striiformis* f. sp. *tritici* (Pst) (Ahmed et al., 2017). Other nsLTPs were found to contribute indirectly by regulating the expression of antioxidant enzymes or by transporting lipids that are involved in ROS detoxification (Song et al., 2023; Xu et al., 2018; Zhu et al., 2023; Li et al., 2024; Hsouna et al., 2021).

Finally, nsLTPs are integral to plant defense signaling pathways. Plant hormones, including abscisic acid (ABA), SA, ethylene, and methyl jasmonate (MeJA), have demonstrated involvement in regulating the expression of nsLTP genes (Missaoui et al., 2022). The ability of nsLTPs to influence the synthesis, transport, and signaling of phytohormones such as SA, JA, and ethylene—central regulators of plant defense—can establish a feedback loop that modulates the overall defense response (Chen et al., 2017). Specifically, nsLTPs facilitate the transport of lipid-based secondary messengers, such as phosphatidic acid (PA) and lysophosphatidylcholine (LPC), which are known to activate downstream defense responses (Lee et al., 1997). Furthermore, nsLTPs are key players in SAR, transporting lipid-derived signals in the phloem to uninfected tissues, effectively priming systemic defenses. DIR1 is a well-studied example of a protein that facilitates long-distance signaling within the SAR pathway (Cameron et al., 2016; Lascombe et al., 2008). nsLTPs are pivotal in activating SAR (Carella et al., 2017; Lascombe et al., 2008; Champigny et al., 2013), functioning as carriers for lipid-based signaling molecules, including precursors of JA and SA. While DIR1’s precise cargo and mechanism remain under investigation (Maldonado et al., 2002), this signaling, along with nsLTP activity, contributes to defense-related gene expression (Blein et al., 2002) (potentially through interaction with elicitin receptors (Buhot et al., 2001) and modulation of JA and SA pathways. Furthermore, nsLTPs contribute to the reinforcement of plant membranes during SAR. The SAR-inducing activity of signals such as azelaic acid (AzA), dehydroabietinal (DA), and glycerol-3-phosphate (G3P) are dependent on functional DIR1 (Duan et al., 2024). Collectively, these functions highlight the crucial involvement of nsLTPs in multiple aspects of the plant immune system.

### 6.1 The role of lipids and nsLTPs in plant immunity

Mechanistically, nsLTPs orchestrate plant immunity through a multifaceted strategy. nsLTPs have been shown to play a role in immune responses against fungi, bacteria, and viruses (Wang et al., 2004; Molina et al., 1993; Ji et al., 2024; Zhu et al., 2023; Zhao et al., 2020; Sun et al., 2008). This includes direct antimicrobial actions, such as disrupting pathogen membranes (Missaoui et al., 2022), a process that may involve permeabilization, pore formation (Regente et al., 2005), and interaction with specific lipids like PA (Madni et al., 2020). They also directly inhibit pathogen growth by interfering with crucial metabolic pathways (McLaughlin et al., 2021; Molina et al., 1993; Jiang et al., 2013; Schmitt et al., 2018), including inhibition of glycosidases (Resende et al., 2023). A nsLTP, *Ca-LTP1* isolated from *Capsicum annuum* is able to inhibit *α*-amylase *in vitro* (Bessiatti Fava Oliveira et al., 2025; Kirubakaran et al., 2008), and a nsLTP isolated from *Ginkgo biloba* can function to inhibit the aspartic acid proteinase, pepsin and the cysteine proteinase papain (Sawano et al., 2008). Finally, nsLTPs integrate with other defense pathways, collaborating with ROS signaling and phytohormone signaling. Their antioxidant properties (Aldakhil et al., 2023; Balmant et al., 2021) are integral to maintaining redox homeostasis and preventing excessive ROS-mediated damage.

Lipids are essential for plant immunity, serving diverse roles beyond their structural function in membranes. They form protective barriers such as the cuticle, composed of cutin and waxes, which prevent pathogen entry and water loss, serving as a first line of defense against pathogens (Zhao et al., 2020; Jacq et al., 2017). Additionally, membrane lipids, including phospholipids and sphingolipids, maintain cellular integrity and are crucial for proper cellular function and immune responses (Dodds et al., 2024; Pretorius et al., 2021; Situ et al., 2024; Sarowar et al., 2009; Guo et al., 2022). Lipids also act as signaling molecules, with phosphatidic acid (PA) and diacylglycerol (DAG) being key examples. PA is a lipid second messenger involved in various stress responses, including the activation of defense-related genes and the production of ROS (Yao and Xue, 2018). DAG plays a role in protein kinase activation and downstream defense signaling pathways (Kalachova et al., 2022). The transport of these signaling lipids by LTPs is crucial for modulating defense responses and intercellular communication. Lipids also serve as precursors for antimicrobial compounds, such as oxylipins and phytoalexins, which combat pathogen infection (Cavaco et al., 2021; Kachroo and Kachroo, 2009). Membrane lipids, such as phospholipids and sphingolipids, are essential for maintaining membrane fluidity and integrity, which are critical for proper cellular function and immune responses. Additionally, certain lipids act as precursors for defense compounds, such as oxylipins and phytoalexins, which are antimicrobial compounds produced by plants to combat pathogen infection (Pretorius et al., 2021; Blée, 1998).

nsLTPs directly support these diverse roles, functioning as important components of plant defense against pathogens (Gao et al., 2022; Kader, 1996; Shah and Chaturvedi, 2009). A survey of the literature highlights the complexity of lipid-mediated plant immunity but also the opportunity to better understand plant-pathogen interactions. Advances in understanding pathogen recognition, signaling pathways, pathogenesis-related proteins, and lipid homeostasis, particularly in relation to nsLTPs, offer a framework for designing bioengineered crops with enhanced resistance to pathogens (Dodds et al., 2024).

nsLTPs are also recognized to play critical roles in the symbiotic relationships between plants and microorganisms, a prime example being the role of nsLTPs in legume–rhizobia symbioses (Zhou et al., 2019; Gao et al., 2022; Gasser et al., 2023; Wei et al., 2022; Chen et al., 2023). In *Medicago truncatula* (Barrel Medic), the nsLTP MtN5 displays a dual role by acting as an antimicrobial agent for plant defense while also being essential for promoting symbiotic root nodulation with *Sinorhizobium meliloti* (Pii et al., 2009). The expression level of the *MtN5* gene directly dictates the quantity of root nodules, as silencing the gene reduced nodule formation by 50% while overexpressing it resulted in a threefold increase in nodule formation. A nodule-specific nsLTP in Chinese Milk Vetch (*Astragalus sinicus*), AsE246, was found to play an important role in the symbiosis with nitrogen-fixing bacterium *Mesorhizobium huakuii* (Lei et al., 2014). Similar to the findings for MtN5 in *M. truncatula*, the expression level of *AsE246* was shown to be a key determinant of symbiotic efficacy. Overexpression of *AsE246* promoted an increase in nodule formation while silencing the gene resulted in a suite of symbiotic defects, including reduced nodulation, lower lipid content within nodules, impaired nitrogen fixation, and aberrant symbiosome development. Later, AsE246, was found to directly bind the high temperature protein G (HtpG) from *M. huakuii* (Zhou et al., 2019), impacting the lipid profile of the root nodules. Gasser et al. (2023) provides a recent review of nsLTPs in nitrogen-fixing symbiosis. Interestingly, the role of nsLTPs in common bean (*Phaseolus vulgaris*) and soybean (*Glycine max*) nodule development is not clear. RT-qPCR expression analysis of roots inoculated with rhizobia has shown the upregulation of PvLTPs (*PvLTPd.4*, *PvLTPd.6*, *PvLTPd.10*, and *PvLTPg.11*) and GmLTPs *GmLTPd.1* and *GmLTP1.1* but functional studies are needed (Fonseca-García et al., 2021).

The lipid biology underlying plant defense mechanisms is complex and dynamic. During pathogen challenge, plants rapidly remodel their membrane composition, produce signaling lipids, and mobilize antimicrobial lipid compounds (Pretorius et al., 2021). nsLTPs orchestrate many of these responses by ensuring the appropriate trafficking of lipid molecules to their required locations for membrane repair, signal transmission, or direct pathogen confrontation (Santos-Silva et al., 2023; Boutrot et al., 2008; Blein et al., 2002; Buhot et al., 2001; Cheng et al., 2004; de Oliveira Carvalho and Gomes, 2007; Edqvist et al., 2018).

### 6.2 Antimicrobial activity of nsLTPs

Some nsLTPs exhibit direct antimicrobial activity, functioning as antimicrobial peptides (AMPs) that target and disrupt pathogen membranes (Gao et al., 2022; Santos-Silva et al., 2023; Amador et al., 2021). Molina et al. (1993) provided an early study on the antifungal nature of nsLTPs. nsLTPs purified from barley (*Hordeum vulgare*) (*Cw18* and *Cw21*) and maize (*Zea mays*) leaves (Cw41) showed activity against phytopathogenic bacteria and fungi. *In vitro* assays using a recombinant nsLTP *Ltp 3F1* cloned from wheat (Sumai 3), was shown to have broad antifungal properties, including *Aspergillus* species, *Candida* species, *Fusarium* species, *Rhizoctonia solani*, *Pyricularia oryzae*, *Alternaria* species, *Botrytis cinerea*, *Pythium debaryanum*, *Phytophthora infestans*, and *Magnaporthe poae* (Kirubakaran et al., 2008).

The rice LTP110 protein was shown to directly inhibit fungal spores of *P. oryzae* (Ge et al., 2002). Using site-directed mutagenesis, the authors showed that the Cys50–Cys89 disulfide bridge was not essential for antimicrobial activity (Ge et al., 2003). Enhanced resistance to *Alternaria solani* and *B. cinerea* was observed in transgenic Arabidopsis plants that expressed the *TdLTP4* gene (Safi et al., 2015). Further research found that recombinant TdLTP4 protein had a broad antimicrobial spectrum and was effective against bacteria and fungi, including *S. aureus*, *L. monocytogenes*, *F. oxysporum*, *F. g.* (Hsouna et al., 2021).

McLaughlin et al. (2021) showed that recombinant *AtLTP4.4* expressed in *Pichia pastoris* was able to inhibit *F. g.* in zone of inhibition assays, possibly by disrupting membranes based on its ability to bind and transfer lipids, leading to alterations in membrane permeability and integrity. Antifungal activity was also noted for the recombinant *Brassica rapa* BrLTP2.1 protein when tested against *F. oxysporum* and *P. syringae* pv. *tomato*. Site-directed mutagenesis of select cysteine residues, for example, Cys69 of BrLTP2.1 impacted the antifungal nature of the protein (Schmitt et al., 2018). Recently, a potato (*S. tuberosum* nsLTP, SpLTPa was shown to bind to and disrupt the plasma membrane (PM) of the oomycete pathogen *P. infestans* but not the PM of the potato cells (Chen et al., 2024). The ability of plant LTPs, including *Coffea canephora* Cc-LTP1 and Cc-LTP2, to permeabilize the membranes of *Candida albicans*, a fungal pathogen associated with nosocomial infections, was also observed (Zottich et al., 2011; Bard et al., 2016).

Do nsLTPs specifically interact with fungal membranes? There are multiple lines of evidence that there are direct interactions. The Ha-AP10 nsLTP from common sunflower (*Helianthus annuus*) was the first nsLTP shown to permeabilize fungal cell membranes, specifically inhibiting *F. solani* spore germination (Regente and De La Canal, 2000). Ha-AP10 was subsequently shown, using fluorescent probes and a liposome leakage assay, to interact directly with phospholipids and this produces the fungicidal effect (Regente et al., 2005). Using chitin affinity chromatography, chitin-binding nsLTPs were isolated from *Capsicum chinense* were identified and characterized (Gonçalves et al., 2024b). These proteins were found to have antifungal activity against *Candida* and *Fusarium* (Gonçalves et al., 2024a, c). Chen et al. (2024) found that PI(3,5)P2 and PI(3)P phosphoinositides competitively inhibit the binding of StLTPa to fungal plasma membranes, diminishing the inhibitory effect of StLTPa. Madni et al. (2020) also demonstrated that a nsLTP isolated from eggplant (*Solanum melongena*) can disrupt fungal membranes. They used the SYTOX Green uptake assay, which measures membrane integrity, to show this disruption (characterized as “bleaching” or increased porosity) via the dye entering the fungal cells upon exposure to the nsLTP.

### 6.3 Role of nsLTPs in cuticle development and integrity

The plant cuticle is composed of a complex mixture of lipids, including cutin and waxes, which create a hydrophobic layer that prevents water loss and protects against pathogen entry. The lipids on the plant surface consist of cutin and waxes (very-long-chain fatty acids (VLCFAs) and their derivatives) (Serrano et al., 2014). Cutin consists of a polymeric network formed by C16 and C18 fatty acids, cross-linked through ester bonds. nsLTPs transport cutin monomers and wax to the plant surface for cuticle assembly and deposition (Jacq et al., 2017; Zhao et al., 2020; Missaoui et al., 2022; Nakamura et al., 2016). nsLTPs facilitate the transport and deposition of lipid molecules essential for cuticle formation by binding and transferring various lipids, such as fatty acids, phospholipids, and glycolipids, from their synthesis sites in the endoplasmic reticulum to epidermal cells. This process ensures a continuous supply of lipids necessary for cuticle development and maintenance. Some evidence of this has come from overexpression studies in plants. For instance, *Arabidopsis* plants engineered to overexpress an nsLTP from saltwater cress (*Thellungiella salsuginea*), *TsnsLTP4*, showed an increase in epicuticular wax deposition (Sun et al., 2015). Likewise, a nsLTP in tomato, *SlLTPG3*, was found to play a crucial role in transporting cuticular wax and cutin to the tomato fruit surface, contributing to enhanced cuticle thickness and reduced permeability. Overexpression of *SlLTPG3* resulted in increased cuticular wax and cutin accumulation, leading to delayed fruit softening and an extended shelf life in tomatoes compared to wild-type plants (Wang et al., 2022).

Another notable example is *Arabidopsis* LTPG (Type G nsLTP), which is a glycosylphosphatidylinositol-anchored lipid transfer protein identified as crucial for exporting lipids to the plant surface. It plays a significant role in transporting cutin and wax precursors, thereby contributing to the proper formation and integrity of the cuticle. Research by DeBono et al. (2009) highlighted the importance of *Arabidopsis* LTPG in maintaining cuticle integrity and function. nsLTPs contribute to cuticle development through their hydrophobic cavity, which allows for stable binding and transport of lipid molecules, as well as targeted delivery facilitated by signal peptide sequences that guide these proteins to specific subcellular locations. Disrupting the nsLTP GPI-anchored 1 (*LTPG1*) gene in *Arabidopsis* altered the plant’s cuticular lipid composition and ultrastructure, negatively impacting its immunity as shown by increased susceptibility to fungal infection (*Alternaria brassicicola*) (Lee et al., 2009).

Additionally, nsLTPs play a role in the plant’s defense mechanisms against abiotic stresses such as drought and salinity by reducing water loss and protecting against desiccation (Nakamura et al., 2016). Their antimicrobial properties further reinforce the cuticle’s barrier function against pathogen attack (Kuźniak and Gajewska, 2024). In summary, nsLTPs, including *Arabidopsis* LTPG, are integral to the development and maintenance of the plant cuticle, enhancing the plant’s ability to withstand environmental stresses and pathogen invasion through the continuous transport of lipid molecules required for cuticle formation.

The broad classes of nsLTP1 and nsLTP2 are known to participate in the transport of phospholipids and glycolipids, contributing to the maintenance of membrane integrity and the formation of lipid-based defense barriers (Aldakhil et al., 2023; Gangadhar et al., 2016; Gasser et al., 2023; Jacq et al., 2017; Schmitt et al., 2018). The integrity of the cuticle is directly linked to plant resistance as a compromised cuticle significantly increases susceptibility to both pathogen invasion and dehydration. Studies have revealed that an nsLTP from *Arabidopsis* plays a structural role in maintaining the adhesion between the hydrophobic cuticle and the hydrophilic cell wall (Jacq et al., 2017; Hairat et al., 2018). During fungal infection, fungal cutinases release cutin which can activate defense responses (Serrano et al., 2014; Arya et al., 2021). In rice, the application of cutin monomer 16-hydroxypalmitic acid (HPA) induces *OsLTP5* expression along with other defense genes (Kim et al., 2008), indicating that plants respond to cuticle damage.

### 6.4 Protein binding by nsLTPs: beyond lipid transport

While nsLTPs are traditionally associated with their ability to bind and transport lipids, emerging evidence underscores their versatile role in protein-protein interactions. This protein-binding capability extends beyond simple lipid binding and exchange, offering a direct mechanism for nsLTPs to modulate cellular processes and interfere with pathogen virulence.

A prominent example of nsLTP protein binding involves their interaction with alpha-amylases. Studies have demonstrated that nsLTPs can bind to and inhibit the enzymatic activity of these hydrolytic enzymes (da Silva et al., 2018b; Bessiatti Fava Oliveira et al., 2025; Zottich et al., 2011). This suggests a resistance mechanism against insect alpha-amylases, potentially disrupting their function and thereby inhibiting carbohydrate digestion, which would serve as a potent plant defense mechanism against herbivory (da Silva et al., 2018b; Saxena et al., 2023). Subsequent research using *C. canephora* Cc-LTP1 and a recombinant *Vigna unguiculata* Vu-LTP1 confirmed that nsLTPs cause potent inhibition of insect intestinal alpha-amylases, leading to reduced larval development in the cowpea weevil, *Callosobruchus maculatus* and supporting the role of these nsLTPs as an active defense mechanism against insect attack by disrupting their digestive processes (da Silva et al., 2018a).

The ability of nsLTPs to modulate carbohydrate metabolism could also play a crucial role in plant stress responses to other threats, such as fungal or bacterial challenge, by influencing the availability of energy and building blocks needed for defense or recovery. A recent study found a potential link between nsLTPs, rice germination rates, and carbohydrate levels in seeds. CRISPR knockouts of *OsLTPL23* negatively impacted rice germination rates and resulted in significantly lower starch levels and higher soluble sugar levels in the edited seeds, the authors hypothesizing that *OsLTPL23* may have alpha-amylase inhibitor activity (Li et al., 2023). Further research is needed to understand the exact role of alpha-amylase inhibition by nsLTPs in plants and if there is a role in disease resistance, specifically how this might affect the availability of sugars crucial for both plant defense and pathogen growth.

More recently, the ability of nsLTPs to directly interact with and modulate pathogen-derived proteins has garnered significant attention. Although a mechanism was not identified, antiviral and antiproliferative activities have been demonstrated for a nsLTP derived from bunch-flowered daffodil (*Narcissus tazetta*) (Ooi et al., 2008). However, more recent work has shown how nsLTPs exert antiviral action. For instance, the cowpea *Vigna unguiculata* LTP1 has been shown to bind to and inhibit the proteolytic activity of the cysteine protease encoded by the CPMV (Ji et al., 2024). This interaction directly interferes with a key viral protein necessary for replication and spread, showcasing a novel antiviral defense mechanism mediated by nsLTPs. The implications of this protein-binding capacity extends beyond viral defense. It is plausible that nsLTPs can interact with other pathogen-derived proteins, such as bacterial or fungal effectors, thereby disrupting their virulence functions (Guo et al., 2022). Exploring the diversity of pathogen proteins targeted by nsLTPs is crucial for understanding the full scope of their contribution to plant immunity. Cataloging the contents of nsLTPs in plants using proteomic and lipomics would help better understand the scope of nsLTP involvement in plant disease response.

### 6.5 Lipid dynamics and signaling in plant immunity: the orchestrating role of nsLTPs

Lipids are dynamic participants in plant immunity, functioning beyond structural roles to generate potent signaling molecules during plant-pathogen interactions (Li et al., 2024). The lipid landscape itself is a dynamic battleground where plants modify lipids for defense, while pathogens exploit them for survival. Upon perception of microbial threats, plants rapidly remodel their lipidome, producing signaling lipids such as phosphatidic acid (PA), lysophosphatidic acid (LPA), oxylipins like jasmonic acid (JA) and 12oxo-phytodienoic acid (OPDA), and sphingolipids such as ceramides (Kuźniak and Gajewska, 2024; Pretorius et al., 2021). These lipid mediators activate diverse pathways, including ROS production, calcium signaling via phosphoinositides, and programmed cell death, collectively shaping robust defense responses. Sphingolipids, like ceramides, induce programmed cell death (PCD), and glycosyl inositolphosphoryl ceramides (GIPCs) contribute to membrane structure and are known to be bound to nsLTPs (Berkey et al., 2012; Gonzalez-Klein et al., 2021). Phosphoinositides (PIs), such as PI4P and PI(4,5)P2, serve as precursors for second messengers like IP3, regulating calcium signaling (Boss and Im, 2012). Additionally, fatty acid derived signals like N-acylethanolamines (NAEs) modulate stress responses (Chapman, 2004; Blancaflor et al., 2014; Hu et al., 2023). These lipid mediators initiate complex signaling cascades, ultimately leading to defense gene expression.

nsLTPs emerge as central regulators of these lipid-mediated defenses, contributing to antimicrobial lipid delivery, membrane reinforcement, lipid signal transduction (e.g., PA, LPC), and long-distance signaling (e.g., DIR1 in SAR) (Santos-Silva et al., 2023; Amador et al., 2021; Chen et al., 2017; Salminen et al., 2016). They facilitate the targeted movement of signaling lipids, reinforce membrane barriers, and orchestrate long-distance immune communication, notably through SAR (Chen et al., 2017; Finkina et al., 2016; Li et al., 2024). The Arabidopsis DIR1 protein exemplifies this function, mediating the movement of lipid-based systemic signals (Carella et al., 2017; Champigny et al., 2013; Lascombe et al., 2008). Beyond mere transport, nsLTPs actively amplify signaling cascades by mobilizing lipid messengers, including PA and lysophosphatidylcholine (LPC). By controlling the spatial and temporal distribution of these lipids, nsLTPs integrate localized and systemic immune responses.

nsLTPs operate within a broader lipid signaling network through coordinated interactions with lipid kinases, phosphatases, and membrane transporters. By delivering lipid substrates to diacylglycerol kinases and phosphoinositide kinases, nsLTPs potentiate lipid signal amplification, while their collaboration with lipid phosphatases ensures signal resolution (Lev, 2010). In parallel, they complement energy-dependent export mediated by ATP-binding cassette (ABC) transporters, particularly during defense-induced membrane remodeling (Roston et al., 2012). At membrane contact sites (MCS), LTPs contribute to lipid exchange between organelles, linking lipid homeostasis with stress responses (Lahiri et al., 2015; Michaud and Jouhet, 2019; Prinz et al., 2020; Zhang et al., 2022). Thus, nsLTPs and LTPs serve not merely as passive shuttles but as dynamic integrators of lipid signaling, tightly coordinating immune activation and cellular resilience.

### 6.6 Mechanisms of action of nsLTPs in plant defense

nsLTPs play diverse roles in plant defense, as illustrated by the following specific examples. *A. thaliana* DIR1, a nsLTP, is crucial for SAR by facilitating long-distance transport of lipid-based signals (Champigny et al., 2013). *Medicago sativa* MsLTP1 exhibits broad-spectrum antimicrobial activity, potentially via a pore-forming mechanism in pathogen membranes (Barashkova et al., 2023). AtLTP4.4 enhances resistance to Fusarium head blight through antifungal and antioxidant activities (McLaughlin et al., 2021). *Nicotiana benthamiana* NbLTP1 boosts immunity against tobacco mosaic virus (TMV) by upregulating SA biosynthesis and downstream signaling components like NPR1 (Zhu et al., 2023).

nsLTPs interact with pattern recognition receptors (PRRs), triggering pattern-triggered immunity (PTI). They are also involved in activating mitogen-activated protein kinase (MAPK) pathways, crucial for defense signaling, partly by transporting lipid-based secondary messengers like phosphatidic acid (PA). Furthermore, nsLTPs integrate with hormonal signaling (SA, JA, ET), enhancing SA biosynthesis and signaling and modulating JA and ET pathways.

The expression of nsLTP genes is regulated by biotic and abiotic stress factors. PAMP recognition by PRRs initiates signaling cascades that activate defense-related genes, including nsLTPs. Key transcription factors (WRKY, MYB, NAC) bind to specific cis-regulatory elements (e.g., W-box, MYB-box) in nsLTP gene promoters. Post-translational modifications, such as glycosylation and phosphorylation, are also important. Glycosylation can enhance nsLTP stability, activity, and localization, while phosphorylation can alter conformation, affecting interactions and activity.

The nsLTPs often contain N-terminal signal peptides and are directed to specific cellular locations like the extracellular apoplast (via the secretory pathway), endoplasmic reticulum, the chloroplast, or vacuoles (Boutrot et al., 2008; Liu et al., 2010; Wang et al., 2012; Nishimura et al., 2008; Chiu et al., 2020). The presence of numerous nsLTP genes raises questions about functional redundancy. While some nsLTPs show overlapping functions in lipid binding and transport, others have specialized roles in cuticle formation, pathogen defense, or reproduction, potentially providing robust defense. Some nsLTPs exhibit direct antimicrobial activity, while others are crucial for SAR by facilitating long-distance transport of lipid signals or maintaining cuticle integrity. The redox-sensitive nsLTP, LTP-II, was shown to be important for guard cell closure in response to the bacterial protein flg22 (Balmant et al., 2021).

SAR is a long-lasting, broad-spectrum immune response triggered by an initial localized pathogen attack. Following local infection, mobile signals are generated and transported to distal tissues, priming them for enhanced defense upon subsequent pathogen exposure. Lipids, lipid-derived molecules, and lipid-associated proteins, particularly nsLTPs, are key mediators of this systemic immune communication.

Among nsLTPs, Arabidopsis DIR1 is a well-characterized example essential for SAR establishment (Champigny et al., 2013; David et al., 2021; Lascombe et al., 2008; Carella et al., 2017). DIR1 facilitates the movement of lipid-based signals through the plant vascular system. Loss of DIR1 function impairs systemic defenses despite normal local responses. Structural and biochemical studies suggest DIR1 carries lipid molecules, likely glycerolipids or phospholipid-derived messengers, crucial for priming distal tissues. While the exact cargo is still under investigation, candidates include lipid derivatives like azelaic acid (AzA) and dehydroabietinal (DA) (Champigny et al., 2013; Lascombe et al., 2008; Carella et al., 2017; David et al., 2021).

Beyond DIR1, other lipid-associated proteins contribute to SAR. AZI1 (Azelaic Acid Induced 1), an LTP-like protein from the hybrid proline-rich protein (HyPRP) family, is implicated in amplifying SAR signaling, potentially by facilitating azelaic acid mobilization (Priya Reddy and Oelmüller, 2024; Gao et al., 2022). AZI1 functions in concert with DIR1, suggesting cooperative action of multiple lipid carriers for robust signal fidelity. The transport of lipid signals by nsLTPs likely occurs through the apoplast and phloem, enabling rapid, energy-efficient dissemination of immune signals.

At the molecular level, SAR involves transcriptional reprogramming, including systemic upregulation of PR genes and increased SA biosynthesis (Vidhyasekaran, 2015). nsLTPs likely interface with these pathways by delivering lipid signals that trigger SA accumulation and by reinforcing membrane and cell wall integrity, enhancing overall stress resilience. Thus, through lipid transport and signal integration, nsLTPs act as key orchestrators of SAR, linking local pathogen recognition to global plant-wide immune readiness.

### 6.7 nsLTPs: versatile proteins with ROS scavenging potential

ROS are highly reactive molecules produced as byproducts of normal cellular metabolism. While ROS are essential signaling molecules in various physiological processes, including defense, excessive accumulation of ROS leads to oxidative stress, causing damage to lipids, proteins, and DNA (Mittler, 2017; Shetty et al., 2008; Wang et al., 2024). nsLTPs mitigate oxidative stress during pathogen attack through direct ROS scavenging or by indirectly upregulating antioxidant genes (McLaughlin et al., 2021, 2015; Yang et al., 2023; Gangadhar et al., 2016; Xu et al., 2018; Wang et al., 2014; Balmant et al., 2021; Hsouna et al., 2021; Safi et al., 2015). In *Nicotiana benthamiana*, overexpression of the type-I nsLTP, *NbLTP1*, activated genes related to ROS scavenging and enhanced resistance to TMV (Zhu et al., 2023). Under thermal stress, tobacco plants overexpressing *NtLTPI.38* exhibited a significant upregulation of genes encoding antioxidant enzymes and thermal stress-related proteins (Song et al., 2023). Similarly, in *NtLTP4* overexpression lines, several important ROS-scavenging enzyme encoding genes, such as *SOD*, *APX*, *CAT*, and *GST*, dramatically increased (Xu et al., 2018). Overexpression of *NtLTP25* significantly increased the enzyme activities of CAT, GST, APX, and SOD, as well as the transcription levels of their encoding gene (Li et al., 2024).

The ROS scavenging activity of nsLTPs has profound implications for plant immunity. By neutralizing ROS, nsLTPs mitigate oxidative damage, protecting cellular components and ensuring the proper function of essential cellular processes. Furthermore, because ROS also act as signaling molecules, nsLTPs can fine-tune ROS levels, influencing the activation of defense pathways and preventing excessive, damaging oxidative stress. Ultimately, by scavenging ROS, nsLTPs enhance the overall stress tolerance of plants, allowing them to better cope with adverse environmental conditions, including pathogen attack. Understanding the mechanisms of their ROS scavenging activity provides valuable insights for improving plant resistance to both biotic and abiotic stresses.

nsLTPs contribute significantly to the plant’s antioxidant defense system. Several mechanisms underlie their ROS scavenging activity. First, the cysteine-rich nature of nsLTPs provides abundant free thiol groups (Wu et al., 2011). These thiol groups can, theoretically, directly interact with and neutralize ROS, such as H_2_O_2_ and superoxide radicals . Second, nsLTPs impact the degree of lipid peroxidation, a chain reaction initiated by ROS that severely damages cellular membranes (Priya Reddy and Oelmüller, 2024; Levesque-Tremblay et al., 2009). For instance, overexpression of *NtLTP25* in tobacco significantly reduced the degree of lipid peroxidation, as indicated by a reduction in malondialdehyde (MDA) levels in the leaves (Li et al., 2024). By stabilizing lipid membranes, nsLTPs may prevent the propagation of lipid peroxidation and protect cellular integrity (Song et al., 2023).

Several examples illustrate the ROS-scavenging capabilities of nsLTPs. Barley LTP1 exhibits strong antioxidant activity, effectively scavenging ROS and protecting cells from oxidative damage (Cai et al., 2019; Stanislava, 2007; Wu et al., 2011). CmnsLTP6.9, from Chinese chestnut, regulates ROS scavenging and remodels lipid profiles, contributing to stress tolerance (Xiao et al., 2023). Similarly, tobacco NtLTPI.38 displays antioxidant capacity, suggesting its involvement in ROS detoxification (Yang et al., 2023). To better understand how the sugar beet responses to abiotic stress, quantitative redox proteomics (iodoTMTRAQ) was used to identify and quantify redox posttranslational modifications (PTMs). Several proteins were identified to be chemically reduced during salt stress, including a nsLTP (A0A0K9RNM7), a novel discovery (Li et al., 2021a). Another example is the LTP-II, which was found to be redox-responsive in response to flg22 during stomatal closure and played a role in plant resistance to *Pseudomonas syringe pv. tomato* DC3000 (Balmant et al., 2021).

How might the cysteines in nsLTPs function to scavenge or otherwise impact ROS accumulation and thereby provide some measure of resistance against oxidative stress? Molecular details from the study of the serine protease inhibitor (serpin) superfamily protein maspin in mouse mammary cells may provide useful information (Mahajan et al., 2013). Maspin is rich in redox-sensitive cysteine residues, containing a total of eight, similar to that found in nsLTPs. It was shown that structurally exposed cysteine thiols were oxidized in the presence of sulfenic acid and that the protein binds to glutathione S-transferase (GST). Another research group, following up on that work, showed that oxidized maspin increases GST, which may lead to the inhibition of oxidative stress-induced ROS generation (Yin et al., 2005). These compelling studies provide a powerful framework for investigating nsLTPs in plants and their potential to modulate ROS. A critical question emerges: does oxidized nsLTP, analogous to maspin, bind to GST in plants, thereby directly influencing its activity and the plant’s capacity to manage oxidative stress? If so, that might help explain why glutathione levels were found to be higher in transgenic *Arabidopsis* overexpressing *AtLTP4.4* and why ROS levels were significantly lower upon exposure to oxidizing agents such as trichothecenes and H_2_O_2_ (McLaughlin et al., 2015). Other nsLTPs may contribute indirectly to ROS accumulation by regulating the expression of antioxidant enzymes or by transporting lipids that are involved in ROS detoxification (Song et al., 2023; Xu et al., 2018; Zhu et al., 2023; Li et al., 2024; Hsouna et al., 2021).

## 7 Genetic engineering of nsLTPs for enhanced plant disease resistance

Genetic engineering offers a powerful strategy for leveraging the inherent disease-protective capabilities of nsLTPs to enhance plant resistance against a broad spectrum of pathogens. Enhanced disease resistance in transgenic plants has been achieved by strategically modifying non-specific lipid transfer proteins (nsLTPs). These modifications include modulating expression levels, altering functional properties, and controlling spatiotemporal expression patterns. Heterologous expression has proved useful in the study and application of nsLTPs. For example, expression of the nsLTP gene *LJAMP2* from motherwort in transgenic poplar trees significantly enhanced their resistance to the fungal pathogens *Alternaria alternata* and *Colletotrichum gloeosporioides* (Jia et al., 2010). Overexpression of *Triticum durum TdLTP2* in *A. thaliana* enhanced resistance against fungal pathogens *Aspergillus niger*, *F. graminearum*, *B. cinerea*, and *A. solani*. Transgenic Arabidopsis expressing barley *LTP2* showed reduced necrotic effects caused by infection by *Pseudomonas* (Molina et al., 1993). Transgenic *Arabidopsis* overexpressing pepper *CALTP1* showed resistance to *P. syringae* and *Botrytis cinerea* (Diz et al., 2011). Expression of *NbLTP1* using a 35S promoter in tobacco enhanced resistance to tobacco mosaic virus (TMV) (Zhu et al., 2023).

Several key approaches can be pursued to enhance disease resistance with nsLTPs. Overexpression of native nsLTP genes, often driven by strong constitutive promoters (e.g., CaMV 35S, maize ubiquitin) or pathogen-inducible promoters, can significantly increase resistance. Two nsLTP genes, *nsLTP4.4* and *nsLTP4.5* were identified from a screen of an activation tagged *A. thaliana* population for resistance to trichothecin, a type B trichothecene in the same class as deoxynivalenol (DON). Overexpression of *nsLTP4.4* provided resistance to Tcin in *Arabidopsis* (McLaughlin et al., 2015). Overexpression of the tomato *SlLTPG1* gene enhanced resistance to *Botrytis cinerea* (Liu et al., 2024). Introduction of heterologous nsLTP genes from other plant species or even non-plant organisms can confer novel defense capabilities. An example is the introduction of the *Arabidopsis AtLTP4.4* gene into wheat, which enhanced resistance to *F.g* (McLaughlin et al., 2021). nsLTP overexpression studies cited in this review are shown in **Table 4**. Creation of chimeric nsLTP variants, combining functional domains or structural modules from different nsLTPs, offers the potential to generate proteins with enhanced or broadened antimicrobial properties. Rational design and protein engineering techniques are crucial for optimizing these chimeric nsLTPs. Targeted expression and subcellular localization using tissue-specific promoters and subcellular targeting signals allows for precise control over where and when nsLTPs are active, minimizing potential off-target effects and maximizing their efficacy. Finally, genome editing technologies, particularly CRISPRCas9, enable precise modification of endogenous nsLTP genes, potentially enhancing their expression or function without the introduction of foreign DNA.

**Table 4:** Summary of nsLTP overexpression studies and resulting phenotypes in plants.

nsLTP Gene	Gene Source (Species)	Host Plant	Promoter	Stress/Pathogen Target	Observed Phenotype
*AtLTP4.4*	*Arabidopsis thaliana*	Wheat and *Arabidopsis*	35S and Expressed in purified from *Komagataella pastoris*	*Fusarium graminearum*	Enhanced resistance, ROS scavenging, lipid remodeling McLaughlin et al. (2021, 2015)
*BrLTP2.1*	*Brassica rapa*	*Arabidopsis*	35S	*F. oxysporum*, *P. syringae*	Antifungal activity, redox-sensitive function Schmitt et al. (2018)
*NbLTP1*	*Nicotiana benthamiana*	*N. benthamiana*	35S	Tobacco mosaic virus (TMV)	Increased SA biosynthesis, enhanced immunity Zhu et al. (2023)
*TdLTP4*	*Triticum durum*	*Arabidopsis*	35S	*Alternaria solani*, *Botrytis cinerea*	Enhanced fungal resistance Safi et al. (2015)
*HvLTP2*	*Hordeum vulgare*	*Arabidopsis*	Expressed in and purified from *E. coli* BL21	Necrotrophic fungi (unspecified)	Reduced necrosis, antifungal activity Hsouna et al. (2021)
*TaLTP4.4*	*Triticum aestivum*	*Arabidopsis*	35S	*Fusarium graminearum*	Membrane disruption, antifungal activity McLaughlin et al. (2015)
*NtLTP25*	*Nicotiana tabacum*	*Tobacco*	35S	Salt, drought stress	Reduced MDA, lipid peroxidation, improved tolerance Li et al. (2024)
*StLTP10*	*Solanum tuberosum*	*Potato*	35S	*Phytophthora infestans*	Increased expression of genes related to ROS scavenging and defense, stomatal closure, wound-induced protein kinase (WIPK) interaction Wang et al. (2021)
*CmnsLTP6.9*	*Castanea mollissima*	*Chestnut* or *Arabidopsis*	35S	ROS and heat stress	Enhanced tolerance, lipidome remodeling Xiao et al. (2023)

A summary of representative genetic engineering studies where the overexpression of specific nsLTP genes led to enhanced plant resilience. The examples showcase that driving high levels of nsLTP expression, often using a strong constitutive promoter like CaMV 35S, can confer heightened resistance against a variety of biotic stresses, including fungal pathogens such as *F.graminearum* and viruses like the TMV. The studies highlight both native expression within the source species and heterologous expression in different host plants, such as introducing a wheat nsLTP into Arabidopsis. In addition to pathogen resistance, the table also includes examples where nsLTP overexpression improved tolerance to abiotic challenges, such as salt and drought stress, often by enhancing the plant’s ROS-scavenging capabilities and reducing lipid peroxidation.

nsLTP overexpression has also been observed to improve abiotic stress resistance. Overexpression of native nsLTPs driven the CaMV 35S promoter in tobacco has increased salt and drought stress resistance (Xu et al., 2018). Overexpressing the Chinese chestnut (*Castanea mollissima*) protein CmnsLTP6.9L in *Arabidopsis* enhanced tolerance to both osmotic and drought stress (Xiao et al., 2023). Increased reactive oxygen species (ROS)-scavenging enzyme activity (SOD and POD) was recorded. Similarly, Xu et al. (2018) overexpressed the native *NtLTP4* in *N. tabacum* to reveal enhanced resistance to salt and drought stresses.

Despite the promise, several challenges and limitations must be addressed. Allergenicity is of significant concern, as nsLTPs are known allergens (e.g., peach Pru p 3 and wheat Tri a 14) (Egger et al., 2010; Gonzalez-Klein et al., 2021; Ruano-Zaragoza et al., 2021; Safi et al., 2019). The low molecular mass and high thermal and proteolytic stability of nsLTPs enable this class of proteins to maintain their tertiary structure and provide a means for the proteins to reach the immune system in a biologically intact form, thereby eliciting an immunoglobulin E (IgE)-mediated hypersensitivity reaction in sensitized individuals. Efforts have been made to generate hypoallergenic nsLTPs in the context of immunotherapy (Gómez-Casado et al., 2013) but these modifications would need to be tested for their impact on nsLTP function (Scheurer and Schülke, 2018). Rigorous allergenicity assessments are essential to ensure the safety of engineered crops for human consumption (Hoffmann-Sommergruber, 2002; Singh and Bhalla, 2008; Salcedo et al., 2007). Pathogen evasion is another critical consideration; pathogens may evolve mechanisms to overcome nsLTP-mediated resistance, necessitating continuous monitoring and development of new strategies.

Deployment strategies can utilize either traditional transgenic approaches by introducing nsLTP genes from other species, or CRISPR-based genome editing, allowing for precise modifications of the endogenous genes. CRISPR offers advantages in terms of precision and potentially fewer regulatory hurdles. To maximize effectiveness and durability, nsLTP-based strategies can be combined with other control methods. Combining nsLTPs with other AMPs, such as defensins or thionins, can broaden the spectrum of antimicrobial activity. Combining nsLTPs with genes that enhance membrane repair and homeostasis (e.g, *AtCHL* for damaged chloroplasts; Levesque-Tremblay et al., 2009) can improve the plant’s ability to maintain cellular integrity during pathogen attack. Integrating nsLTPs into broader integrated pest management (IPM) strategies, which include cultural practices, biological control, and judicious use of chemical treatments, offers a holistic approach that reduces reliance on any single method.

## 8 LTPs and membrane contact sites

Membrane contact sites (MCSs) are dynamic junctions where organelles connect through protein tethers, serving as critical hubs for the non-vesicular transfer of lipids, ions like calcium, and small molecules between organelles (Scorrano et al., 2019; Michaud and Jouhet, 2019). These specialized zones facilitate efficient molecular transfer by allowing organelles to closely appose without fusing, forming essential microdomains (Lahiri et al., 2015). This inter-organelle communication is vital for maintaining cellular homeostasis (Michaud and Jouhet, 2019; Wang et al., 2023; Man et al., 2024). While extensively studied in yeast and mammalian systems for their roles in calcium signaling, mitochondrial dynamics, and lipid metabolism (Michaud and Jouhet, 2019; Banday et al., 2022), the understanding of MCS function in plants remains limited, representing a significant knowledge gap.

LTPs are key players at MCSs, binding lipids within hydrophobic pockets and facilitating their transfer across the aqueous cytosol between membranes (Michaud and Jouhet, 2019). The close proximity of organelle membranes at MCSs significantly shortens the diffusion distance for LTPs, thereby accelerating lipid transfer (Zhang et al., 2022). LTPs are highly conserved and classified by lipid-binding specificity, including sphingolipid-, sterol-, and phospholipid-transfer proteins. Notable examples include oxysterol-binding protein (OSBP) and its related proteins (ORPs), which mediate sterol and phosphoinositide transfer, and ceramide transfer protein (CERT), involved in ceramide transfer between the ER and Golgi (Renna et al., 2024; Nakatsu and Kawasaki, 2021; Kumagai and Hanada, 2019). In plants, LTPs at MCSs contribute significantly to both lipid homeostasis and defense mechanisms by facilitating the transfer of various lipids, including phospholipids and glycolipids (Michaud and Jouhet, 2019).

In plants, MCSs are crucial for efficient lipid trafficking and signaling, supporting cellular homeostasis and mediating responses to both biotic and abiotic stresses. The presence of plant-specific organelles, such as plastids, introduces unique MCS functionalities linked to photosynthesis, nutrient assimilation, and stress responses (Yao et al., 2023). During pathogen attack, MCSs are hypothesized to be critical for the rapid exchange of signaling molecules that trigger defense responses (Michaud and Jouhet, 2019; Paul and Tiwari, 2023). For instance, they likely mediate the transfer of lipids like PA and phosphatidylinositol phosphates (PIPs), which are critical in plant defense. However, the precise molecular mechanisms and specific proteins involved in these plant MCS-mediated processes is not well understood. Recently, LaBrant (2025) identified chloroplast MCS proteins using a transient expression screen in *N. benthamiana*. The *Arabidopsis* nsLTP GPI-anchored 20 (LTPG20, At3g22620) was found to localize to chloroplasts in this study. Additional research will be necessary to verify LTPG20 functions in chloroplast lipid trafficking at MCSs but this type of research highlights efforts to catalog nsLTPs and LTPs at plant MCSs.

Mechanisms of stress resistance involving MCSs are also being discovered in mammalian systems. Under stress conditions, mammalian (HeLa) mitochondria can generate oxidized lipids, which are efficiently transferred to the ER via LTPs localized at mitochondria-ER MCSs (Sassano et al., 2022; Shiiba et al., 2025). These contact sites facilitate the non-vesicular transfer of peroxidized lipids, enabling rapid responses to oxidative stress (Shiiba et al., 2025). Similar LTP mechanisms may operate in plants during pathogen-induced stress; for example, certain virulence factors, such as trichothecene mycotoxins, are known to induce mitochondrial stress (Bin-Umer et al., 2014). It remains to be determined if damaged plant mitochondria utilize a similar mechanism to manage oxidized lipids during biotic stress. This is an emerging aspect of plant biology with likely important roles in plant responses to pathogens.

## 9 Future research directions

Understanding the dynamic interplay of nsLTPs in lipid transport and signaling offers valuable insights into their application in developing disease-resistant crops. Biotechnological strategies that leverage the functional capabilities of nsLTPs hold significant potential to enhance plant defense mechanisms. Moreover, emerging research reveals that nsLTPs serve roles far beyond immunity, including oxidative stress mitigation and long-distance signaling, positioning them as key molecular players in the broader landscape of cellular lipid metabolism.

To unlock the full potential of nsLTPs in plant defense and enable their effective deployment in agricultural biotechnology, several strategic research directions must be pursued. Central to this is the detailed elucidation of their structural and functional properties. High-resolution techniques such as X-ray crystallography and cryo-electron microscopy are essential to visualize nsLTPs in complex with diverse lipid ligands, thereby advancing our understanding of ligand specificity and binding mechanisms. Biochemical and biophysical characterization, using methods such as isothermal titration calorimetry (ITC) or surface plasmon resonance (SPR), can yield critical data on lipid binding affinities and kinetics. In parallel, site-directed mutagenesis can identify key residues involved in lipid binding, transfer, and potential antimicrobial activity, guiding efforts in protein engineering and synthetic biology.

A comprehensive understanding lipid dynamics and trafficking is also vital. Mass spectrometry-based lipidomics can profile shifts in lipid composition during pathogen attack, illuminating how specific lipids are mobilized and potentially interact with nsLTPs. This approach can also delineate how pathogen-derived effectors disrupt lipid signaling networks or alter membrane composition to suppress host defenses. Integrating lipidomics with functional genomics and proteomics will further clarify the downstream consequences of pathogen-mediated lipid manipulation. Furthermore, utilizing lipidomics in organelle trafficking studies may reveal the nuanced roles nsLTPs play in inter-organelle lipid exchange, particularly under stress conditions.

Further investigation into the antifungal and antiviral properties of nsLTPs, their crosstalk with other defense signaling pathways (e.g., salicylic acid, jasmonic acid, ethylene), and the evolutionary strategies pathogens employ to circumvent nsLTP-mediated immunity are also critical areas of future inquiry. Mechanistic studies are needed to determine how nsLTPs exert antifungal and antiviral effects, identify targets, and evaluate the spectrum of their protective activity. Understanding how pathogen effectors directly target or evade nsLTP function can inform the design of more durable, resistance-conferring interventions.

Several promising avenues exist for biotechnological applications. The rational design of engineered nsLTP variants guided by structural and functional insights offers significant promise. Variants with enhanced lipid-binding affinity, broader antimicrobial spectra, or increased biochemical stability can be tailored for targeted expression in specific tissues (e.g., epidermis, vascular tissue) or subcellular compartments (e.g., apoplast, chloroplast). Such targeted deployment can optimize defense responses while minimizing potential fitness trade-offs. Combinatorial strategies that integrate nsLTP-based interventions with traditional resistance genes or biocontrol agents may yield robust, multi-layered plant protection systems.

In addition, the role of nsLTPs in membrane repair and oxidative stress management warrants focused investigation, particularly in relation to chloroplast integrity under pathogen-induced damage. Studies examining nsLTP interactions with specific chloroplast lipids and antioxidant pathways may uncover novel mechanisms of stress resilience. These integrated efforts will be instrumental in developing crops with superior adaptability and disease resistance. Rigorous field validation and a thorough evaluation of biosafety and regulatory considerations will be essential before widespread adoption of nsLTP-engineered crops. If successful, such advances will contribute meaningfully to agricultural sustainability and global food security.

## 10 Conclusion

nsLTPs are emerging as pivotal regulators of plant defense, orchestrating lipid transport, membrane remodeling, and signaling cascades in response to pathogenic threats. Their multifunctional nature, which encompasses antimicrobial activity, ROS scavenging, and modulation of systemic defense pathways, which are summarized in **Table 5**, underscores their vital role in maintaining cellular homeostasis during biotic stress.

**Table 5:** Key findings on nsLTPs in plant defense.

Key Finding/Function	Description/Details
Role in Plant Immunity	nsLTPs are PR proteins vital for plant defense, managing lipid dynamics during pathogen infection. They enhance plant resilience.
Antimicrobial Activity	Act as direct antimicrobial agents against various pathogens (fungi, bacteria, viruses). Mechanism involves disrupting pathogen membranes.
ROS Scavenging	Protect plant cells by scavenging damaging ROS, reducing oxidative stress during pathogen attack. This can be a direct action via cysteine residues.
Cuticle Formation	Essential for building and maintaining the plant cuticle by transporting lipid precursors like cutin monomers and waxes.
Defense Signaling	Modulate key defense signaling pathways. Transport lipid-based signals (e.g., PA, JA precursors) for local and systemic defense responses, including SAR. DIR1 is a notable nsLTP in SAR.
Lipid & Ligand Binding	Characterized by a versatile hydrophobic cavity allowing them to bind a wide range of lipids (phospholipids, fatty acids, etc.) and other hydrophobic molecules.
Protein Interactions	Some nsLTPs can bind directly to other proteins, such as inhibiting pathogen or plant enzymes (e.g., alpha-amylases, viral proteases).
Membrane Contact Sites	LTPs (and perhaps nsLTPs) operate at MCSs, facilitating inter-organelle lipid transfer crucial for stress responses and homeostasis.
Biotechnological Potential	Modifying nsLTP expression (e.g., overexpression) in plants shows promise for engineering enhanced resistance to diseases and abiotic stress. Allergenicity of nsLTPs demands careful attention and testing.

A consolidated overview of the principal functions and characteristics of nsLTPs in the context of plant defense, summarizing the major themes discussed throughout this review. The multifunctional nature of nsLTPs is highlighted, detailing direct protective roles such as antimicrobial activity and ROS scavenging, structural contributions to the plant cuticle, and nsLTP involvement in orchestrating local and systemic defense signaling pathways. Additionally, the versatile ligand-binding abilities of nsLTPs are presented, the capacity for protein-protein interactions, and the potential for improving crop resilience through biotechnology.

Equally important is their function in sustaining lipid homeostasis. nsLTPs facilitate membrane integrity, organelle crosstalk particularly at MCS, and dynamic lipid signaling, all of which are essential for robust immune responses. By shuttling lipids between compartments, they ensure both structural stability and signaling precision.

Genetic manipulation of nsLTP expression has demonstrated promise in conferring broad-spectrum resistance across a range of crop species. Exploring the interplay between nsLTPs and lipid signaling in plant disease contexts presents a compelling avenue for next-generation crop improvement strategies.

A multidisciplinary approach, which integrates lipidomics, advanced imaging, systems biology, and plant pathology will be critical to fully exploit the potential of nsLTPs. This integrated framework can inform the design of novel, sustainable crop protection strategies that are both scientifically rigorous and commercially viable. Continued innovation and research are imperative to establish nsLTPs as central components of modern agricultural biotechnology and to address the growing global demand for resilient, high-yield crops.

## Supplementary material

Supplementary Table 1 Chronological comparison of the major classification systems developed for the plant nsLTP superfamily, summarizing the key literature from (Kader, 1996) to Huang et al. (2023). This timeline illustrates the evolution of the defining criteria used to categorize these proteins, beginning with early systems based on fundamental physical properties like molecular mass. The table tracks the progression to more sophisticated, multifaceted approaches that incorporate sequence homology, the precise spacing of the 8CM, and genomic features such as intron positioning. It also documents the recent expansion of the nsLTP family to include novel lineages discovered in algae, which was made possible by adapting broader bioinformatic search parameters. This comprehensive overview serves as a foundational reference for understanding the various nsLTP types and the scientific rationale behind their classification as discussed throughout this review.

Supplementary Table 2 A detailed overview of the nsLTP superfamily across the plant kingdom, organized by major evolutionary lineages. The table provides a species-specific look at the number of nsLTPs identified, the major types present, and their key structural and functional characteristics. Important evolutionary patterns are highlighted, such as the existence of specific nsLTP types (like Type D) in early land plants like bryophytes, the general absence of Types I and II in those early lineages, and the recent discovery of a distinct nsLTP lineage in green algae that challenges previous assumptions about their origins. This comparative summary illustrates the diversification of the nsLTP family as plants adapted to new environments and pathogens.
