# Supplementary material for: Roles of non-specific lipid transfer proteins in plant defense: structural and functional perspectives
Source: Front Fungal Biol. 2025 Sep 16;6:1640465. doi: 10.3389/ffunb.2025.1640465 (PMC12480973; doi:10.3389/ffunb.2025.1640465)
Supplement: Supplementary Table 1 — Chronological comparison of the major classification systems developed for the plant nsLTP superfamily, summarizing the key literature from (Kader, 1996) to Huang et al. (2023). This timeline illustrates the evolution of the defining criteria used to categorize these proteins, beginning with early systems based on fundamental physical properties like molecular mass. The table tracks the progression to more sophisticated, multifaceted approaches that incorporate sequence homology, the precise spacing of the 8CM, and genomic features such as intron positioning. It also documents the recent expansion of the nsLTP family to include novel lineages discovered in algae, which was made possible by adapting broader bioinformatic search parameters. This comprehensive overview serves as a foundational reference for understanding the various nsLTP types and the scientific rationale behind their classification as discussed throughout this review. [file DataSheet1.pdf]

Supplemental Table 1: **Comparison of Plant nsLTP Classification Systems and Key Characteristics**

Reference	No. of Types	Type/Group Names	Key Defining Criteria	MM Range (kDa)	pI Range	8CM Notes
Kader, 1996	2	Type I, Type II	Molecular mass	Type I (9), Type II (7)	Usually 8.5 to 12	C-Xn-C-Xn-CC-Xn-CXC-Xn-C-Xn-C backbone; different cysteine pairing patterns for types I and II.
Boutrot et al., 2008	9 (I-IX) + nsLTPY	Types I-IX, nsLTPY	Sequence similarity, intervals of eight cysteine residues	Gen. 6.5–10.5. Rice examples: I (8.9–12.3), II (6.9–8.1), III (6.7–6.8), IV (8.3–8.9), V (9.4–9.9), VI (8.7–10.9)	Majority basic (3.92–12.68 overall). Rice examples: I (3.92–12.20), II (7.06–12.14), III (7.84), IV (7.84–10.65), V (9.69–12.05), VI (4.48–10.18)	Consensus C-Xn-C-Xn-CC-Xn-CXC-Xn-C-Xn-C pattern. Specific spacing variations per type.
Wang et al., 2012	5 (I-V) + Type X	Types I-V, Type X (also A, B, C, D)	Patterns of 8 highly-conserved cysteine residues, sequence similarity	Type I (8.0–15.0), Type II (6.1–9.3), Type III (7.2–8.9), Type IV (9.3–10.5), Type V (9.3–10.8)	Type I (3.67–12.30), Type II (4.50–12.02), Type III (4.50–6.73, 9.52–10.17), Type IV (8.48–12.31), Type V (4.75–5.27, 9.82)	Prosite-styled patterns for Type I and II. E.g., Type I: C X2 V X5-7 C [V, L, I] × Y [L, A, V] X8-13 CC × G X12 D × [Q, K, R] X2 CXC X16-21 P X2 C X13-15C. Table 2 details flanking residues.
Edstam et al., 2011/2016	10 (1, 2, C-K)	Types 1, 2, C, D, E, F, G, H, J, K	Sequence similarity, GPI modification site, intron position, spacing between cysteine residues	Not consistently summarized. Ex: *M. polymorpha* Type D (8.1–19.2), Type G (9.1–25.6)	Not consistently summarized. Ex: *M. polymorpha* Type D (3.78–11.26), Type G (3.96–5.98)	Spacing of Cys residues analyzed per type. Often notes intron positions relative to 8CM (e.g., Type D, G often have intron 4 bases downstream of 8th Cys codon).
Fleury et al., 2019	10 (I-VI, VIII, IX, XI, +12 unclassified)	Types I-VI, VIII, IX, XI	New criteria: seq. length (60–150 res. incl. signal peptide), strictly 8 Cys after signal peptide removal, 8CM pattern (C-Xn-C-Xn-CC-Xn-CXC-Xn-C-Xn-C), monodomain proteins (excl. hybrid proline/glycine-rich proteins).	Defined by sequence length criteria (60–150 residues).	Not explicitly summarized by type.	Strict 8CM pattern. Excluded types not satisfying 8CM (e.g., Boutrot's Type VII had only 7 Cys).

Continued on next page

Supplemental Table 1: **Comparison of Plant nsLTP Classification Systems and Key Characteristics** (continued)

Reference	No. of Types	Type/Group Names	Key Defining Criteria	MM Range (kDa)	pI Range	8CM Notes
Huang et al., 2023	New lineage (29 genes)	Algal nsLTPs (sometimes AI, AII)	8CM presence, secretory signal peptides, MM ¡60 kDa, phylogenetic clustering with land plant nsLTPs.	10.36–50.28; mostly 10–25 (82.76%). Gen. larger than land plant nsLTPs (¡10).	Not explicitly summarized. Ex: CrLTP1 pI 5.53, CrLTP2 pI 8.64	8CM pattern with extended spacing (n 8–50). Can have extra N-terminal region upstream of 1st Cys.
