# Supplementary material for: Roles of non-specific lipid transfer proteins in plant defense: structural and functional perspectives
Source: Front Fungal Biol. 2025 Sep 16;6:1640465. doi: 10.3389/ffunb.2025.1640465 (PMC12480973; doi:10.3389/ffunb.2025.1640465)
Supplement: Supplementary Table 2 — A detailed overview of the nsLTP superfamily across the plant kingdom, organized by major evolutionary lineages. The table provides a species-specific look at the number of nsLTPs identified, the major types present, and their key structural and functional characteristics. Important evolutionary patterns are highlighted, such as the existence of specific nsLTP types (like Type D) in early land plants like bryophytes, the general absence of Types I and II in those early lineages, and the recent discovery of a distinct nsLTP lineage in green algae that challenges previous assumptions about their origins. This comparative summary illustrates the diversification of the nsLTP family as plants adapted to new environments and pathogens. [file DataSheet2.pdf]

Supplemental Table 2: **Comprehensive Overview of Plant Non-Specific Lipid Transfer Proteins (nsLTPs)**

Plant Species	nsLTP Type / Lineage	Number of nsLTPs	Key Characteristics	Associated Classification Systems / Notes	References
**General Plant Kingdom**	**nsLTP Superfamily**	Thousands	Generally **small, basic proteins** (molecular mass typically **6.5 to 10.5 kDa**) with a basic isoelectric point (pI). Possess a **conserved 8-cysteine motif (8CM)** (C-Xn-C-Xn-CC-Xn-CXC-Xn-C) with 4 disulfide bonds, forming a **conserved common right-handed superhelix structural fold** and an **internal hydrophobic cavity** for lipid binding/carrying. Exhibit high **thermostability**. Involved in various key roles including **plant development and defense**, membrane stabilization, cell wall organization, and signal transduction.	Constitute a superfamily of related proteins widely distributed across the plant kingdom. Their biological functions, despite extensive characterization, remain largely unclear.	José-Estanyol, Gomis-Rüth & Puigdomènech, 2004; Boutrot et al., 2008; Wang et al., 2012; Liu et al., 2015; Fleury et al., 2019; Huang et al., 2023
**General Plant Kingdom**	**Type I nsLTPs**	Varies; Major type	Mature protein sequence is approximately **90 amino acids** long with a molecular mass of **9 kDa**. Characterized by a **long tunnel-like cavity**. Disulfide bridges are typically linked as C1-C6, C5-C8, C2-C3, and C4-C7. Genes often contain a **single intron**.	One of the two most established nsLTP subfamilies. Identified in 88 species, comprising 391 members in the nsLTPDB. Shared by all species identified to possess nsLTPs.	Douliez et al., 2000; Wang et al., 2012; Liu et al., 2015; Huang et al., 2023
**General Plant Kingdom**	**Type II nsLTPs**	Varies; Major type	Mature protein sequence is approximately **70 amino acids** long with a molecular mass of **7 kDa**. Characterized by **two adjacent hydrophobic clefts**. Disulfide bonds are typically linked as C1-C5, C6-C8, C2-C3, and C4-C7. Genes generally **do not contain introns**.	One of the two most established nsLTP subfamilies. Identified in 23 species, comprising 102 members in the nsLTPDB. Shared by all species identified to possess nsLTPs.	Douliez et al., 2000; Wang et al., 2012; Liu et al., 2015; Huang et al., 2023
**General Plant Kingdom**	**Type C nsLTPs**	Varies	Characterized by a comparatively **lower pI (4–7)**. In *Arabidopsis*, they are specifically expressed in the anther tapetum and are involved in the assembly of sporopollenin in the exine layer of the pollen wall. Genes typically carry **one intron**.	Overlaps with Boutrot et al.'s Type III classification. Part of the classification system proposed by Edstam et al. (2011).	Edstam et al., 2011; Liu et al., 2015; Huang et al., 2023

Continued on next page

Supplemental Table 2: **Comprehensive Overview of Plant Non-Specific Lipid Transfer Proteins (nsLTPs)** (continued)

**Plant Species**	**nsLTP Type / Lineage**	**Number of nsLTPs**	**Key Characteristics**	**Associated Classification Systems / Notes**	**References**
**General Plant Kingdom**	**Type D nsLTPs**	Varies	Considered the **earliest established nsLTP type** in plant evolution. Genes typically carry **one intron located four nucleotides downstream of the last Cys codon of the 8CM**.	Expressed in liverworts, mosses, and vascular plants. Overlaps with Boutrot et al.'s Type V and Type VIII classifications. Has been retained from bryophytes to angiosperms with a close evolutionary relationship.	Edstam et al., 2011; Liu et al., 2015; Huang et al., 2023
**General Plant Kingdom**	**Type G nsLTPs (LTPG)**	Varies	Encode proteins with an **extra Glycosylphosphatidylinositol (GPI) anchoring motif at the C-terminus**. Functions are related to **cuticle deposition** and **cell wall organization**. Expression can be restricted to aerial parts of plants (e.g., AtI/OsI module for cuticular wax). Other modules (AtII/OsII for suberin, AtIII/OsIII for sporopollenin) exist.	Common in liverworts and present in *Arabidopsis thaliana* and rice. Overlaps with Boutrot et al.'s Type VII and Type VIII classifications.	Edstam et al., 2011; Kim et al., 2012; Edstam et al., 2013; Liu et al., 2015; Huang et al., 2023
**Green Algae (Chlorophytes, Charophytes)**	**Algal nsLTPs (New Lineage)**	Mainly 1-2 per haploid genome (e.g., *K. nitens*: 2; *C. reinhardtii*: 2; except *Coccomyxa sp. C-169* with 8)	Characterized by a **molecular mass of 10.36–50.28 kDa**, predominantly 10–25 kDa (82.76%), which is **significantly larger than most land plant nsLTPs** (¡10 kDa). Possess an **extended spacing sequence in the 8CM region**, often enriched with hydrophilic residues. Have **extended N- and C-terminal regions** flanking the 8CM, featuring turn-forming residues like proline and glycine. Genes typically carry **multiple introns located within the 8CM region** (e.g., 4 in *Gonium pectoral*, 5 in *CrLTP1/2*, 6 in *Tetrabaena socialis*), unlike land plants that usually have 0 or 1 intron after the C8 codon. CrLTP2 from *Chlamydomonas reinhardtii* has been shown to bind fatty acids, phospholipids, and sterols.	**Contradicts earlier findings** that nsLTPs are not present in algal genomes. This new lineage suggests nsLTPs may have arisen from a common ancestor of green algae and land plants. They form a distinct mono-clade with land plant nsLTPs in phylogenetic analyses, separate from other 8CM-containing proteins.	Edstam et al., 2011; Liu et al., 2015; Huang et al., 2023

Continued on next page

Supplemental Table 2: **Comprehensive Overview of Plant Non-Specific Lipid Transfer Proteins (nsLTPs)** (continued)

**Plant Species**	**nsLTP Type / Lineage**	**Number of nsLTPs**	**Key Characteristics**	**Associated Classification Systems / Notes**	**References**
**Bryophytes (e.g., *Marchantia polymorpha*, *Physcomitrella patens*)**	**Diverse nsLTP Types (excluding Type I & II)**	*M. polymorpha*: 14; *P. patens*: 40	**LTP1 and LTP2 types are generally not found** in bryophytes or lycophytes. **Type D and G are dominant** and are hypothesized to be among the earliest evolved nsLTPs in land plants. *Physcomitrella patens* uniquely features **multidomain nsLTPs** with two or three connected 8CMs.	Gene copy number is typically lower than in vascular plants, potentially suggesting less redundancy. Bryophytes serve as valuable model systems for investigating cuticle function and assembly.	Edstam et al., 2011; Edqvist et al., 2016; Edstam et al., 2018; Fleury et al., 2019; Huang et al., 2023
**Lycophytes (e.g., *Selaginella moellendorffii*)**	**Diverse nsLTP Types (excluding Type I & II)**	43	Includes Type D (19 genes) and a unique Type H (6 genes). **LTP1 and LTP2 types are generally not found** in lycophytes.	Type H was previously reported as restricted to *S. moellendorffii*.	Edstam et al., 2011; Edqvist et al., 2016; Edstam et al., 2018;
